# Transcriptome and Biochemical Analysis Reveals That Suppression of GPI-Anchor Synthesis Leads to Autophagy and Possible Necroptosis in *Aspergillus fumigatus*

**DOI:** 10.1371/journal.pone.0059013

**Published:** 2013-03-18

**Authors:** Jianghong Yan, Ting Du, Wan Zhao, Thomas Hartmann, Hua Lu, Yang Lü, Haomiao Ouyang, Xuejun Jiang, Lei Sun, Cheng Jin

**Affiliations:** 1 State Key Laboratory of Mycology, Institute of Microbiology, Chinese Academy of Sciences, Beijing, China; 2 Institute of Biophysics, Chinese Academy of Sciences, Beijing, China; Saint Louis University, United States of America

## Abstract

Previously, it has been shown that GPI proteins are required for cell wall synthesis and organization in *Aspergillus fumigatus*, a human opportunistic pathogen causing life-threatening invasive aspergillosis (IA) in immunocompromised patients. Blocking GPI anchor synthesis leads to severe phenotypes such as cell wall defects, increased cell death, and attenuated virulence. However, the mechanism by which these phenotypes are induced is unclear. To gain insight into global effects of GPI anchoring in *A. fumigatus*, in this study a conditional expression mutant was constructed and a genome wide transcriptome analysis was carried out. Our results suggested that suppression of GPI anchor synthesis mainly led to activation of phosphatidylinositol (PtdIns) signaling and ER stress. Biochemical and morphological evidence showed that autophagy was induced in response to suppression of the GPI anchor synthesis, and also an increased necroptosis was observed. Based on our results, we propose that activation of PtdIns3K and increased cytosolic Ca^2+^, which was induced by both ER stress and PtdIns signaling, acted as the main effectors to induce autophagy and possible necroptosis.

## Introduction

*Aspergillus fumigatus* is an opportunistic pathogen causing life-threatening invasive aspergillosis (IA) in immunocompromised patients [1], [2]. The crude mortality from IA is 60–90% [3]. This high mortality is due to a poor understanding of *A. fumigatus* and the low efficiency of drug therapies available. A deeper understanding of *A. fumigatus* at the molecular level would help to develop new drugs or new strategies to treat IA.

As the fungal cell wall directly contacts the host cells and provides physical protection against an adverse environment, it has long been treated as an ideal target for drug development. The cell wall of *A. fumigatus* is mainly composed of a covalently connected polysaccharide skeleton (glucans and chitin) that is interlaced and coated with glycoproteins [4]–[6]. Some cell surface proteins are modified at their C-terminus by the addition of a glycosylphosphatidylinositol (GPI) anchor and transported to the plasma membrane and cell wall, where they are directly or indirectly involved in cell wall organization [7]–[14].

GPI anchoring is a conserved post-translational modification in eukaryotes, by which many proteins such as cell surface enzymes, receptors, and adhesion molecules are anchored to the cell membrane. The core structure of the GPI anchor consists of a lipid group, myoinositol, glucosamine (GlcN), several mannose residues and a phosphoethanolamine (EtN) group, which ultimately connects the GPI anchor to the protein via an amide bond. Although the number of mannose groups and the position of the side-chains on the GPI anchors vary widely between species, a common core structure of EtN-Man_3_GlcN-PI is conserved in all GPI-anchored proteins found in protozoa, yeast, plants and mammals. GPI is assembled at the endoplasmic reticulum (ER) in multiple steps catalyzed by the concerted actions of approximately 20 proteins [15]. The first step of GPI anchor synthesis is initiated by the transfer of N-acetylglucosamine (GlcNAc) from UDP-GlcNAc to phosphatidylinositol (PtdIns), which is catalyzed by PIG-A/Gpi3p, a catalytic subunit in the glycosylphosphatidylinositol-N-acetylglucosaminyltransferase (GPI-GnT) complex [15].

GPI anchoring is not essential in mammals at a cellular level as several GPI-deficient cell lines have been established [16]. However, an acquired GPI-anchoring deficiency in haematopoietic stem cells causes paroxysmal nocturnal haemoglobinuria [17], a rare but severe human disease. In contrast to mammals, GPI anchor synthesis is essential in *Saccharomyces cerevisiae* [18]. Previously, we could show that deletion of *afpig-a*, the homologue of the *GPI3*/*pig-A* gene in *A. fumigatus*, leads to a complete blocking of GPI anchor synthesis, cell wall defects, abnormal hyphal growth, aberrant conidiation and attenuated virulence in immunocompromised mice. Although the mutant is viable, it displays a significant increase of cell death [13].

In order to gain a better understanding of the mechanism of increased cell death triggered by deficient GPI anchoring, we constructed a conditional expression mutant of the *afpig-a* gene in this report. Genome-wide transcriptome analysis and biochemical analyses reveal that reduced expression of the *afpig-a* gene led to an increased cell death through autophagic process, and possibly through necroptotic process as well, in *A. fumigatus*.

## Materials and Methods

### Strains and growth conditions

*Aspergillus fumigatus* strain YJ-407 (China General Microbiological Culture Collection Center, CGMCC0386) was maintained on potato glucose (2%) agar slant [19]. *A. fumigatus* strain CEA17 [20], a gift from C. d'Enfert, Institute of Pasteur, France, was propagated at 37°C on complete medium (CM) [21], or minimal medium (MM) with 0.5 mM sodium glutamate as a nitrogen source [21]. Uridine and uracil were added at a concentration of 5 mM when required. Mycelia were harvested from strains grown in CM at 37°C with shaking at 200 rpm. At the specified culture time point, mycelia were harvested and washed with distilled water, then frozen in liquid nitrogen and ground by hand. The powder was stored at −80°C for DNA, RNA and protein extraction. Conidia were prepared by growing *A. fumigatus* strains on solid CM with uridine and uracil (CMU) at 37°C for 36 h. The spores were collected, washed twice with 0.01% Tween 20 in PBS and resuspended in PBS, and its concentration was confirmed by haemocytometer counting and viable counting. Vectors and plasmids were propagated in *Escherichia coli* DH5α (Bethesda Research Laboratories).

### Construction of the conditional *afpig-a* mutant

Plasmid pAL3 containing the *A. nidulans alcA* promoter (P*_alcA_*) and the *Neurospora crassa pyr-4* gene as a fungal selectable marker [22] was employed to construct a suitable vector allowing the replacement of the native promoter of the *afpig-a* with the P*_alcA_*. For this goal, an 853-bp fragment from -52 to +801 of the *A. fumigatus afpig-a* genomic sequence was amplified with primers P1 (5′-GGGGTACCGTTTATCCC ATACTCATCGCGAAG-3′, including a *Kpn*I site) and P2 (5′-GCTCTAGATGA CAACAATTGTGATAGTATCGT-3′, containing an *Xba*I site). The PCR-amplified fragment was cloned into the expression vector pAL3 to yield pALpig-aN and then sequenced. The plasmid pALpig-aN without mutation was transformed into *A. fumigatus* strain CEA17 by PEG-mediated transformation of protoplasts [23] and colonies with uridine/uracil autotrophy were screened on MM plates. The transformant was confirmed by PCR and Southern blotting. For PCR analysis, three pairs of primers (P3 and P4 are for the 1623-bp *afpig-a* gene, P5 and P4 are for the 2023-bp *alcA-afpig-a*, P6 and P7 are for the 1217-bp *pyr-4* fragment) were employed (P3: 5′-ATGGTTTGTGACTTCTTCTTC-3′; P4: 5′-TCATGGGGCAAGACGCTCC TG-3′; P5: 5′-TCGGGATAGTTCCGACCTAGGA-3′; P6: 5′-AAACGCAAATCA CAACAGCCAA -3′; P7: 5′-CTATGCCAGACGCTCCCGG -3′).

For Southern blotting, genomic DNA was digested with *EcoR*I, separated by electrophoresis and transferred to a nylon membrane (Bio-Rad). The 1137-bp amplified internal fragment of the *amp* gene and the 853-bp *afpig-a* N fragment were used as probes. The *amp* probe was amplified with P8 (5′-CTATGCCAGACGCTC CCGG-3′) and P9 (5′-CTATGCCAGACGCTCCCGG-3′). Labeling and visualization were performed using the DIG DNA labeling and detection kit (Roche Applied Science) according to the manufacturer's instruction.

### Construction of the *ΔqutG* mutant

A 1.1-kb upstream region of the *qutG* gene before the ATG was amplified with the primers P10 (5′-GGGGTACCCCCTCTTCGTCTGATACGCTC-3′, containing a *Not*I site) and P11 (5′-CCCAAGCTTGTTAACATTGTGTATGCAGATTGGGA-3′, containing an *Hpa*I site). A 1.3-kb downstream region of the *qutG* gene after the stop codon was amplified with the primers P12 (5′-CCCAAGCTTGTTAACTGATG AATACATTCGTTCTAT-3′, containing an *Hpa*I site) and P13 (5′- GGAATTCCATATGGCGGCCGCACTACAACCTCAGAAGCACTA-3′, containing a *Nde*I/*Not*I site). The upstream and downstream regions were cloned into the relevant sites of the pEGM-T Easy Vector (Promega). The *pyrG* blaster cassette (8.6 kb) was released by the digestion of pCDA14 with *Hpa*I and was cloned into the site between the up- and downstream regions of *qutG*, to yield the deletion construct pAFQutG-pyrG. After digestion with *Not*I, the linearized pAFQutG-pyrG was transformed into strain CEA17 and screened for mutants with uridine and uracil autotrophy.

The transformants were confirmed by PCR and Southern blotting analysis. For PCR analysis, four pairs of primers were employed. Primers P14 (5′-CGCAATCGAGAAG AGATAC-3′) and P15 (5′-AGATACAACCGACTGCCCA-3′) were used to amplify the *qutG* gene. Primers P16 (5′-TGTCTCCTCATCAAGTGTG-3′) and P17 (5′- ATCGTAGATGATTAGGCGGG-3′) were used to amplify the *pyrG* gene. Primers P18 (5′-CAGTACCAGCAGACGTATAGC-3′) and P19 (5′-TGAAGCTCGCG CAGATCAGTTG -3′) were used to amplify the fragment containing the *neo* cassette and the upstream sequence of the *qutG* gene. Primers P20 (5′- GAGTTCTACCGGCAGTGCAAATC-3′) and P21 (5′- ATCGTAGATGATTAGG CGGG-3′) were used to amplify the fragment containing the downstream region of the q*utG* gene and the *neo* cassette.

Genomic DNA was digested with *Xho*I, separated by electrophoresis, and transferred to a nylon membrane (Bio-Rad). The 1.1-kb upstream region of the *qutG* gene was used as the probe. Labeling and visualization were performed using the DIG DNA labeling and detection kit (Roche Applied Science).

### Construction of the PtdIns3K over-expression strain OEpi3k

The ORF of PtdIns3K without its terminator was amplified using primer pairs PtdIns3k-up (5′-GGGTTTAAACATGGAGGCATTCACATTTGC-3′) and PdtIns3k-down (5′-GGGTTTAAACCGCTCTCCAACCCTGCAC-3′) from *A. fumigatus* cDNA. The PCR products were digested with *Pme*I and ligated into plasmid pVG2.2 (a gift from Leiden University) which contains *pyrG* as the selected maker. The plasmid obtained (pVG2.2-*pi3k*) was transformed into the *pyrG^−^* strain CEA17. The PtdIns3K overexpression strain OEpi3k was confirmed by PCR. Using Pr-up (5′-CCTGAAACCCAACCCTAAGA-3′) and Pr-down (5′-TCTTCTGCTGTG AGGTCCTG-3′) as primers, Real-time PCR analysis was carried out to detect an 81-bp fragment of the gene encoding PtdIns3K with TBP as the control.

### Assay for the activity of GPI-GnT toward PtdIns

2×10^8^ spores were inoculated into 200 ml CM media and cultivated at 37°C, 200 rpm for 36 h. Proteins from the wild-type (WT) or mutant were extracted and assayed as previously described [13].

### Detection of GPI anchor and GPI proteins

2×10^8^ spores were inoculated into 200 ml CM media and cultivated at 37°C for 36 h with shaking at 200 rpm. Mycelia were collected and ground by hand. The mycelium powder was suspended with distilled water and centrifugated at 4,000 g. The supernatant containing membrane protein and incellular protein was dried by lyophilization.

To detect the GPI anchor, 1 mg of cell lysate was added to 200 µl of HF pyridine (Sigma) and incubated on ice for about 12 h. 200 µl of 30% (v/v) pyridine were added as control. After digestion, proteins were removed by adding 20 µl of 100% TCA (Sigma) and centrifuged at 13,000 rpm for 10 min. The supernatant was dried in a vacuum drier (Thermo Scientific) and purified by Bond Elut^R^ C18 (Varian) and Sep Cartrodge Carbograph (Dikma) columns. The purified GPI anchor was detected by high performance anion exchange chromatography (HPAEC-PAD, Dionex). Elution was performed at a flow rate of 1 ml/min with 29 mM NaOH.

To detect GPI proteins, 1 mg of cell lysate was dissolved in 200 µl of HF pyridine (Sigma) and incubated on ice for 10 min. The reaction was stopped by the addition of an equal volume of ice-cold H_2_O. After dialysis against H_2_O overnight to remove HF-pyridine, proteins were dried by lyophilization. The protein powder was dissolved in 100 µl ddH_2_O and analysed by SDS-PAGE. Proteins were visulalized by Coomassie brilliant blue R-250.

### Microarray experiments

The *A. fumigatus* DNA microarrays were custom designed using the Agilent eArray 5.0 program according to the manufacturer's recommendations (http://earray.chem.agilent.com/earray/). The chip specification was 8×15K. The genome sequences were downloaded from: http://www.ncbi.nlm. nih.gov/genome?Db = genome&Cmd = Search&Term = txid330879[orgn]. Each gene was represented by one 60-nt oligonucleotide probe, and 358 genes out of the total 9630 genes were replicated 14 times each.

Total RNA was extracted from strains grown in liquid CM at 37°C for 36 h with shaking at 200 rpm by using Trizol (Invitrogen). Total RNA was purified using the Qiagen RNeasy Mini Kit (Qiagen). Two milligrams of RNA was reverse-transcribed into cDNA and then transcribed into cRNA. After purification, 4 mg of cRNA was labeled with Cy3 NHS ester (GE Healthcare) and purified. Hybridization was performed using the Gene Expression Hybridization Kit (Agilent). After fragmentation, 1 mg of cRNA was hybridized at 65°C for 17 h with a rotation at 10 rpm. Then, the arrays were washed twice using wash buffer 1 and 2 from the Gene Expression Wash Buffer Kit (Agilent). Finally, arrays were scanned using an Agilent Microarray Scanner System (G2565BA) (Agilent) with a resolution of 5 µm, 100% and 10% PTM respectively and the two data sets were combined automatically.

The signal intensities were normalized using the Feature Extraction Software (Agilent). Data was analyzed using Genespring Software 5.0. Genes with all signals present (flag = P) were selected for analysis. Experiments were repeated four times. Differentially expressed genes were selected with P≤0.05, FC≥1.5 by T-test methods. Pathways were analysed by the SAS pathway enrichment suite (Shanghai Biotechnology Corporation) using the genes with a fold change of 1.5 or higher. Microarray data obtained in this study has been deposited in Gene Expression Omnibus of the NCBI (Accession Number: GSE42499).

### Phenotypic analysis

Growth of the *A. fumigatus* strains on various media was carried out by inoculation of 10^2^–10^5^ spores of the WT or mutant strain onto solid CM, YEPD or MM supplemented with 0.1 M ethanol, 0.1 M glycerol, 0.1 M threonine, or 1–3% glucose. Plates were incubated at 37°C for 28 h and photographed.

To detect the cell wall defect, a series of 10-fold dilutions (10^7^–10^4^ cells) of spores was spotted onto the solid CM or MM supplemented with 0.1 M ethanol, 0.1 M glycerol, or 0.1 M threonine containing 250 µg/ml Congo red or 250 µg/ml Calcofluor white and incubated at 37°C for 36 h.

For morphology observation, 2×10^8^ spores were inoculated into 200 ml liquid CM and incubated at 37°C with shaking (200 rpm). 100 µl of sample were observed under a differential interference contrast (DIC) microscope (Olympus).

For propidium iodide (PI) staining, 2×10^8^ conidia were inoculated in 200 ml of liquid CM and incubated at 37°C for 24 h with shaking (200 rpm). The mycelia were collected, stained with PI, and then examined under the fluorescene microscope (Zeiss).

For transmission electron microscopy (TEM), the mycelia cultivated in liquid CM at 37°C for 7 h, 12 h, 24 h or 36 h were collected and fixed in 2.5% glutaraldehyde in 0.1 M phosphate buffer (pH 7.0) for 4 h or overnight at 4°C. Cells were fixed in 2.5% glutaraldehyde in 0.1 M phosphate, washed 3 times in 0.1 M phosphate, post-fixed in 1% osmium tetroxyde, incubated for 2–4 h in 0.1 M phosphate, then 15–20 min in methanol 30%, 50%, 70%, 85%, 95% and 100%, respectively, post-fixed in 2% of 30% uranyl acetate-methanol. Cells were rinsed, dehydrated and embedded in Epon 812 for the floating sheet method. The section was examined with a Tecnai Spirit (120 kV) transmission electron microscope (FEI).

### Chemical analysis of the cell wall

Conidia were inoculated into 200 ml liquid CM at a concentration of 10^6^ conidia/ml and incubated at 37°C for 36 h with shaking (200 rpm). The mycelia were harvested, washed with deionized water and frozen at −80°C. Ten milligrams of dry mycelial pad were added to an eppendorf tube containing 50 mM NH_4_HCO_3_ (pH 8.0) and 0.2 g of glass beads (1 mm diameter). The mycelium was disrupted by successively shaking the tube with a Disruptor Genie (Scientific Industries) for five times (5 min each). Then the cell homogenates were centrifuged and washed several times with distilled water. Three independent lyophilized mycelia were used in each test. The experiment was repeated 3 times using mycelia from different cultures.

The cell wall was treated with 1 M KOH and incubated at 70°C for 30 min to release glycoprotein and α-glucan. Alkali-soluble materials were acidified with acetic acid to pH 5.0 and the precipitated α-glucans were collected by centrifugation and washed with water. Glycoprotein in the supernatant was precipitated with 2 volums of ethanol, washed twice with 64% ethanol and dissolved in distilled water. Glycoprotein concentration was determined using the Lowry protein assay [24]. Monosaccharides were liberated from glycoprotein by acid hydrolysis (6 M HCl at 100°C for 2 h) and separated on a CarboPac PA1 anion-exchange column, equipped with an Amino Trap guard column (Dionex). Elution was performed at room temperature at a flow rate of 1 ml/min with 18 mM NaOH.

Alkali-insoluble materials were washed with distilled water several times and digested in 6 M HCl at 100°C for 2 h to release monosaccharides from β-glucan and chitin. After digestion, HCl was evaporated and the residues were dissolved in 0.2 ml distilled water [25]–[27]. The amounts of α-glucan and β-glucan were estimated by measuring released glucose using the phenol/sulfuric acid method [28]. Chitin content was determinated by measuring the N-acetylglucosamine released after digestion using the method described by Lee et al. [29].

### Real-time PCR

Total RNAs were extracted using TRIZOL (Invitrogen). The cDNA synthesis was performed with 5 µg RNA using the RevertAid™ First Strand cDNA Synthesis Kit (Fermentas). Primers P22 (5′-CTCAACCAGGGGGAGTTGAA-3′) and P23 (5′-AG ATATCGAACTCCAGTCCG-3′) were used to amplify a 117-bp fragment of the *afpig-a*, and primers TBP-5′ (5′-CCACCTTGCAAAACATTGTT-3′) and TBP-3′ (5′-TACTCTGCATTTCGCGCATG-3′) were used to amplify an 80-bp fragment of the gene encoding RNA polymerase I and III transcription factor complex component Tbp (AFUA_3G10120). To exclude contamination of cDNA preparations with genomic DNA, primers were designed to amplify regions containing one intron in the gene. The PCR reaction was done with SYBR® *Premix Ex Taq*™ (Takara) in a reaction mixture containing 8.8 µl cDNA, 10 µl SYBR® *Premix Ex Taq*™ (2×), 0.4 µl ROX Reference Dye (50×) and 0.2 µM of each pair of primers. Thermal cycling conditions were 50°C for 2 min and 95°C for 10 min, followed by 40 cycles of 95°C for 15 s, 60°C for 60 s. A triplicate of samples was tested in each assay and each experiment was repeated 3 times. RNAs extracted from a separate batch of mycelia were used for confirmation of the differentially expressed genes. The primers used are listed in Table S1.

### Determination of caspase activity

Proteins were extracted by grounding mycelia in liquid nitrogen, resuspending the powder in ice-cold lysis buffer (50 mM Hepes, pH 7.4, 1 mM DTT, 0.5 mM EDTA, and 0.1% (v/v) Chaps), and centrifugation at 1,500 g for 10 min [30]. The caspase activities of the supernatant against substrates for caspase-1, -3, and -8 were determined using a fluorescent assay based on the cleavage of a AMC (7-amino-4-methylcoumarin) dye from the C-terminal of specific peptide substrates (Caspase Fluorescent (AMC) Substrate/Inhibitor *QuantiPak*™) (BioMol International).

### Determination of surface-exposed phosphatidylserine

Mycelia were harvested by filtration onto sterile muslin, washed with 100 ml protoplast buffer (0.1 M phosphate buffer, pH 7, 1 M NaCl, and 10 mM MgCl_2_), and resuspended in protoplast buffer to 40 mg/ml wet weight. Enzyme from *Trichoderma harzianum* (Sigma) was added to a final concentration of 3 mg/ml and incubated on a rotary shaker (80 rpm) for 1 h (until approximately 30% of the mycelia had been protoplasted). Protoplasts were recovered by filtration through four layers of lens tissue, centrifuged at 1,500 g for 5 min and resuspended in an equal volume of regeneration buffer (0.1 M phosphate buffer, pH 7, 0.9 M sorbitol) [30].

The percentage of protoplasts in which phosphatidylserine had been exposed on the surface was determined using the Annexin V-FITC Apoptosis Detection Kit (Sigma) and observed under epifluorescence (excitation 494 nm and emission 520 nm). To determine membrane integrity, 100 µl of the protoplast suspension were centrifuged (1,500 g for 10 min), resuspended in 0.5 ml binding buffer (50 mM Hepes, 750 mM NaCl, 12.5 mM CaCl_2_, and 5 mM MgCl_2_), and incubated for 15 min before adding 10 µl propidium iodide (30 µg/ml in phosphate-buffered saline, 137 mM NaCl, 2.7 mM KCl, 4.3 mM Na_2_HPO_4_ **.**7H_2_O, and 1.4 mM KH_2_PO_4_). Protoplasts were incubated for 15 min in the dark and analysed under epifluorescence (525 nm excitation and 590 nm emission).

### Determination of DNA fragmentation

Protoplasts were subjected to Terminal Deoxynucleotidyl Transferase-mediated dUTP Nick End Labelling (TUNEL) Staining using Fluorescein FragEL™ DNA Fragmentation Detection Kit (Calbiochem). Stained protoplasts (TUNEL-positive) were detected using fluorescence microscopy.

### Effect of chemical compounds on growth of the mutant

A series of 10-fold dilutions (10^3^–10^5^ cells) of spores was spotted onto solid CM containing 1‰ DMSO, 0.2 µM wortmannin or 25 µM necrostatin-1. The plates were incubated at 37°C for 36 h and photographed. For the effects of FK506 and rapamycin on the germination of the mutant, spores were inoculated into liquid CM containing 1‰ DMSO, 5 µM FK506 or 10 nM rapamycin at a concentration of 10^6^ conidia/ml and incubated at 37°C with shaking (200 rpm) for different amounts of time, samples were detected under a DIC microscope (Olympus). Triplicates were used in each assay and the experiment was repeated 3 times.

### Effect of ER stress related agents on the growth of the mutant

A series of 10-fold dilutions (10^7^–10^4^ cells) of spores was inoculated into 1 ml liquid CM containing 1‰ DMSO, 4 mM DTT or 5 µg/ml tunicamycin (Sigma) and incubated at 37°C for 36 h. The experiment was repeated 3 times.

### Ca^2+^ detection

2×10^8^ spores were inoculated into 200 ml liquid CM and incubated at 37°C for 5 h or 24 h with shaking (200 rpm). The cells were collected by centrifugation,washed 3 times with PBS, and resuspended in fresh CM containing 5 µM Fluo 3-AM (Sigma). Subsequently, the cell suspensions were incubated at 37°C for 1 h in the dark and then washed twice with PBS to remove the extracellular Fluo 3-AM. Fresh CM was added to the cells and the cell suspension was incubated at 37°C for another 30 min. Finally, the fluorescence was detected under a fluorescence microscope (Zeiss) with a 506 nm excitation light and the fluorescence emission was at 525 nm. The experiment was repeated 3 times.

### Western blotting

2×10^8^ spores were inoculated in 200 ml liquid CM and incubated at 37°C for 36 h with shaking (200 rpm). To isolate the extracellular proteins, 2 ml freshly prepared 2% (w/v) sodium deoxycholate was added to the media (1/100 in volume). Extracellular proteins from the culture supernatant were precipitated with 20 ml of 100% trichloroacetic acid at 4°C for 30 min, and collected by centrifugation (15,000 g at 4°C for 15 min). The precipitate was washed 3 times with acetone and dried.

Total cellular proteins were extracted from mycelia with lysis buffer (100 mM Tris-HCl,0.01% SDS,1 mM DTT, pH 7.5). Proteins in the supernatant were collected by centrifugation (13,000 rpm at 4°C for 10 min).

For preparation of cytosolic and membrane proteins, mycelia were ground by hand in liquid nitrogen and suspended in 200 mM Tris-HCl (pH 8.0) containing 50 mM EDTA and protease inhibitor cocktail (Sigma) at 4°C for 30 min. The homogenate was centrifuged (4,000 g) at 4°C for 10 min. The supernatant was then ultra-centrifuged (45,000 rpm) at 4°C for 1 h. The cytosolic proteins in the supernatant and the membrane proteins in the precipitate were collected separately. Protein Concentration was determined by Bradford protein assay [31].

Fifty micrograms of cellular proteins were separated by 10% SDS-PAGE and transformed to a PVDF membrane (Millipore). The proteins were detected using anti-phosphorylate-specific Erk antibody (Cell Signaling), anti-RIP1 antibody (BD Biosciences) or anti-RIP3 (Abcam) antibody. As control, an anti-RAS antibody (Cell Signaling) was used.

Equal amounts of extracellular, cytosolic, or membrane proteins from the WT or mutant were separated by 10% polyacrylamide gels, transferred to a PVDF membrane (Millipore), and detected with custom-designed antibodies against the Ecm33, Gel1, or Gel4, which were developed in specific pathogen free (SPF) rabbits using synthesized peptides (Ecm33: TITISSQSDADGYSSC; Gel1: CPAKDAPNWDVDN DALPA; and Gel4: AKWEASNKLPPSPNSELC) (B&M Company).

### Identification of the target of the anti-RIP3 antibody in *A. fumigatus*

Proteins extracted from the mutant were incubated with Protein A Sepharose™ beads (GE Healthcare) at 4°C for 1 h with agitation. The mixture was centrifuged at 3,000 rpm, at 4°C for 3 min to discard the beads. The supernatant was incubated with commercial RIP3 (Abcam) antibody at 4°C for 4 h with agitation. Beads were added into the mixture containing the antigen-antibody complex and incubated at 4°C for another hour. The mixture was centrifuged at 3,000 rpm, at 4°C for 3 min to remove the supernatant. Proteins bound to beads were released by washing with TBST buffer and separated on 10% polyacrylamide gels and stained with Coomassie brilliant blue R-250. For in-gel digestion of proteins, the protein band was cut out, destained in 50% (vol/vol) acetonitrile (Sigma) in 40 mM NH_4_HCO_3_, pH 8.4, dehydrated with 100% acetonitrile, and dried using a SpeedVac drying apparatus (Thermo Scientific). The proteins were reduced with 10 mM dithiothreitol (Sigma) in 40 mM NH_4_HCO_3_ at 56°C for 60 min and then alkylated for 45 min at room temperature with 55 mM iodoacetamide in 40 mM NH_4_HCO_3_. The gel pieces were washed with 40 mM NH_4_HCO_3_ for 15 min, dehydrated with 100% acetonitrile, and dried using a SpeedVac (Thermo Scientific). The gel slices were rehydrated with 12. ng/µl of mass spectrometry (MS)-grade Trypsin Gold (Promega) in 40 mM NH_4_HCO_3_. The protease-generated peptides were extracted with 0.1% (v/v) formic acid in 20 mM NH_4_HCO_3_ and subjected to LC-MS detection.

Capillary LC-ESI-ion trap (IT) mass spectrometry (LCQ Deca XP^plus^ mass spectrometer, Thermo-Finnigan) in the MS/MS mode was used to analyze the amino acid sequences of the tryptic peptides. The peptide mixture was dissolved in 20 µl 0.1% formic acid and loaded onto the pre-equilibrated ThermoHypersil C-18 column (180 µm×100 mm, Biobasic). The flow rate was maintained at 120 µl/min before the flow split, and at 1.5 µl/min after the split. The gradient was started at 5% acetonitrile in 0.1% formic acid for 20 min, then ramped to 50% acetonitrile in 80 min, and finally ramped to 95% acetonitrile for additional 20 min. The temperature of the ion transfer tube was set at 200°C. The spray voltage was set at 3.3 kV and the normalized collision energies were set at 35% for MS/MS. The MS/MS data was acquired in the data dependent scan mode including four scan events: one full-range MS scan, and three MS/MS scans on the three most intense precursor masses (as determined by Xcalibur mass spectrometer software in real time) from the single parent full scan. Dynamic mass exclusion windows were used. MS spectra for all samples were measured with an overall mass/charge (m/z) range of 400–2000.

The sequences of the un-interpreted collision-induced dissociation (CID) spectra were identified by SEQUEST algorithm incorporated into the Thermo-Finnigan Bioworks software (Version 3.1). These idealized mass spectra were weighted largely with *b* and *y* ions, i.e. fragments resulting from the amide linkage bond from the N and C termini, respectively. The SEQUEST analysis was performed using the *A. fumigatus* FASTA protein database downloaded from NCBI. The SEQUEST search results were initially assessed by examination of the Xcorr (cross correlation) and ΔCn (delta normalized correlation) scores. As a general rule, an Xcorr value greater than 3.75 for triply charged, 2.2 for a doubly charged, and 1.9 for singly charged ions, and ΔCn greater than 0.1 was accepted as a positive identification.

## Results

### Construction of the conditional *afpig-a* mutant

**Figure 1:**
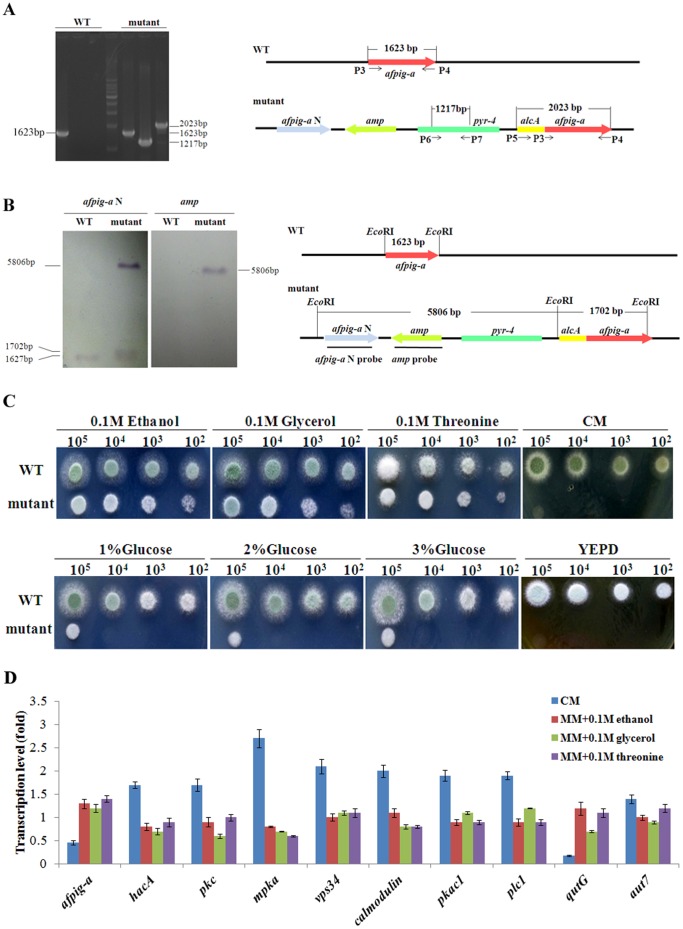
Construction of the conditionally express *afpig-a* mutant. In A, PCR confirmation of the mutant was carried out as described under Materials and Methods; in B, a Southern blotting was carried out by probing the *EcoR*I-digested genomic DNA with an *afpig-a N* probe or an *amp* probe; and in C, 10^2^–10^5^ spores of the wild-type (WT) and the mutant strain were inoculated onto CM, YEPD, or MM supplemented with ethanol, glycerol, threonine, or glucose. The plates were incubated at 37°C for 28 h; in D, the expression of 10 selected genes was detected by Real-time PCR. RNAs were extracted using mycelia cultivated in different media at 37°C for 36 h with shaking (200 rpm). The experiment was repeated 3 times.

A conditional expression mutant was constructed by replacing the native promoter of the *afpig-a* gene with P*_alcA_*, a strictly regulated promoter that can be induced by ethanol, glycerol or threonine and repressed completely on YEPD medium [22], [32]. To this end, the plasmid pALpig-aN, which contains the *pyr-4* gene, P*_alcA_*, and 3′ truncated version of the *afpig-a* gene, was employed in a transformation of the *A. fumigatus* CEA17 strain to generate a strain carrying the P*_alcA_-pig-a* fusion gene by homologous recombination. Five transformants were obtained and only one transformant was confirmed to be correct. PCR analysis confirmed that a 1217-bp fragment of *pyr-4* and a 2023-bp fragment of P*_alcA_-afpig-a* were amplified from the genomic DNA of the mutant, while no such fragments were amplified from the WT DNA (Fig.1A). Southern blotting analysis of the *EcoR*I-digested genomic DNA of the mutant confirmed a correct integration. A 5806-bp fragment containing *pyr-4*, *amp* and *afpig-aN* was detected, while no such fragment was found in the WT (Fig.1B). These results demonstrated that the promoter of the *afpig-a* gene was replaced by P*_alcA_* in the mutant.

As shown in Fig.1C, the growth of the mutant on MM supplemented with ethanol, glycerol, or threonine was similar to wild type growth and inhibited on YEPD, CM, or MM containing glucose. When the mutant was grown on CM, the expression of the *afpig-a* gene was 45.9±3.9% of the WT. Meanwhile, the GPI-GnT activity that transfers GlcNAc to phosphatidylinositol (PtdIns) was reduced to 52.8% in the mutant and the amount of GPI anchor produced in the mutant was only 78.1% of that in the WT. Therefore, CM was chosen as the repressive medium in order to investigate the transcriptome and strain phenotypes in this study.

To exclude the possibility that secondary mutations were introduced during the construction of the mutant strain, the expression patterns of 10 selected genes in the WT and conditional expression mutant were anlaysed. As shown in Fig.1D, expression of these genes in the mutant strain was similar to that of the WT under inducing conditions, but differentially expressed in the mutant under repressive condition.

### Microarray analysis of the conditional *afpig-a* mutant

The RNA extracted from the mutant grown in CM at 37°C for 36 h was assayed with a genome-wide microarray chip. As a result, 3274 genes were differentially expressed (P<0.05), either induced or repressed at least 1.5-fold (Table S2) (NCBI Accession Number: GSE42499). These differentially expressed genes belong to 88 pathways (Table S3). Enriched pathways include amino acids metabolism, sugar metabolism, lipid metabolism, protein synthesis and degradation, electron transport respiratory chain, lipid peroxidation, peroxidation, autophagy, MAPK signaling, and PtdIns signaling. Some of the differentially expressed genes were confirmed by Real-time PCR (Table 1).

**Table 1:** RT-PCR analysis of the differentially expressed genes in the mutant.

Locus tag	Protein	pathway	Fold change	
			Microarray	RT-PCR
AFUA_4G13720	MAPK MpkA/Slt2	Cell wall integrity	1.5	2.7
AFUA_5G11970	PKC PkcA/Pkc1	Phosphatidylinositol signaling/MAPK signaling pathway	ND	1.7
AFUA_8G03930	Hsp70 chaperone HscA	Protein processing in endoplasmic reticulum	2.7	2.8
AFUA_2G04620	Hsp70 BiP/Kar2	protein export	2.0	3.7
AFUA_6G14130	UBE2G1	Ubiquitin-mediated proteolysis	3.1	2.0
AFUA_1G05970	GRR1	ubiquitin ligase complex F-box/Ubiquitin-mediated proteolysis	3.2	2.1
AFUA_2G01170	Gel1	Cell Wall synthesis	11.8	12.0
AFUA_6G11390	Gel2	Cell Wall synthesis	4.8	4.3
AFUA_5G09100	MAPK Mpkc	Osmolarity sensing	−2.7	−2.6
AFUA_5G08670	PtdIns3K	Phosphatidylinositol signaling	1.5	2.1
AFUA_1G13250	Plc1	Phosphatidylinositol signaling	2.0	1.9
AFUA_4G10050	Calmodulin	Phosphatidylinositol signaling	2.2	2.0
AFUA_2G08470	GTP binding protein Bud4	Cell wall integrity	1.6	2.2
AFUA_2G05740	Rho GTPase ModA/Cdc42	MAPK signaling pathway	1.5	2.5
AFUA_2G02760	Msg5	MAPK signaling pathway	1.7	3.0
AFUA_2G12200	PKA PkaC1	cAMP-dependent protein kinase catalytic subunit PkaC1	ND	1.9
AFUA_2G16520	PLD	Glycerophospholipid metabolism	−2.2	−2.6
AFUA_1G11600	QutG	Phosphatidylinositol metabolism	−5.6	−5.7
AFUA_6G09165	Apg12/Atg12	Autophagy	2.0	1.6
AFUA_5G08170	Aut1/Atg3	Autophagy	6.6	5.1
AFUA_4G10800	S6e	ribosome	3.3	3.2
AFUA_3G12300	L22e	ribosome	2.5	2.5
AFUA_3G13840	Imp2	protein export	−2.1	−1.7
AFUA_2G17560	Arp2	Biosynthesis of unsaturated fatty acids	−2.5	−4.5
AFUA_1G15670	TilA	Melanin synthesis	−4.5	−3.4
AFUA_1G14290	Lsm7	RNA degradation/spliceosome	2.7	2.1

The real-time PCRs were performed on a separate batch of RNA which was different from the RNA used in the microarray experiments. ND, not detected.

### Cell wall defect and compensatory mechanism in the mutant

**Figure 2:**
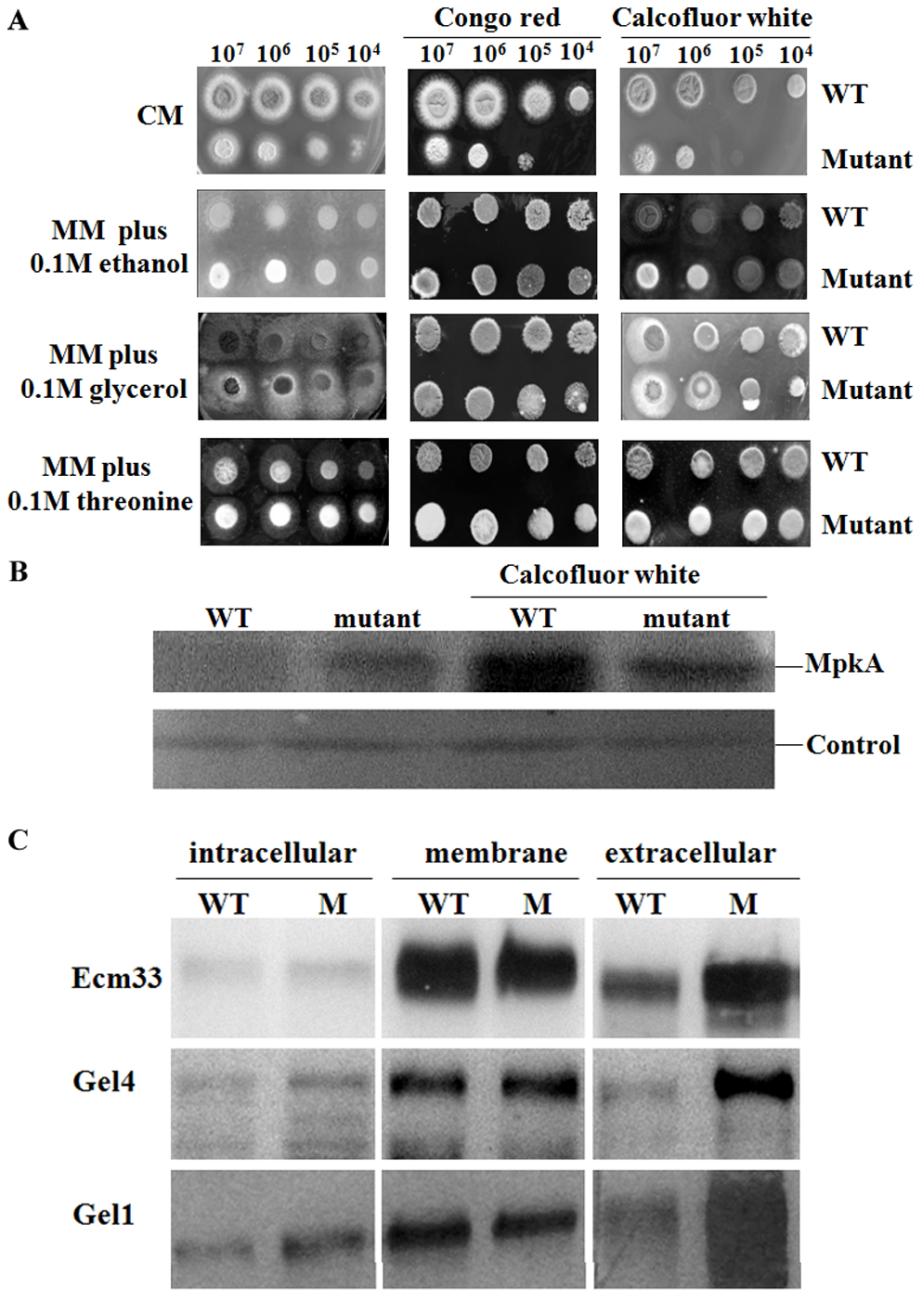
Cell wall defect, activation of MpkA and ER stress in the conditional *afpig-a* mutant. In A, a series of 10-fold dilutions (10^7^–10^4^ cells) of spores was spotted onto solid CM or MM supplemented with 0.1 M ethanol, 0.1 M glycerol, 0.1 M threonine containing 250 µg/ml Congo red or 250 µg/ml calcofluor white. The plates were incubated at 37°C for 36 h and photographed; in B, a Western blotting was carried out using an anti-phospho-p44/42 MAPK (Erk1/2) (Thr202/Tyr204) antibody. 50 µg of total protein from the wild-type (WT) or mutant strain cultivated in CM at 37°C for 36 h were extracted, separated by SDS-PAGE, transferred to a PVDF membrane and then detected with the antibody. As a control, an anti-RAS antibody was used to detect an unrelated protein; and in C, a Western blotting was carried out using an antibody against the *A. fumigatus* Ecm33, Gel4, or Gel1.

As shown in Fig.2A, the mutant displayed an increased sensitivity to Congo red and calcofluor white under repressive conditions. Cell wall component analysis confirmed a decrease of â-glucan and glycoprotein content in the mutant (P<0.05) (Table 2). These results indicate a cell wall defect in the mutant. Transcriptome analysis revealed that 16 GPI protein genes were suppressed at least 1.5-fold in the mutant, including the genes encoding putative 1,3-β-glucanosyltransferase Gel3, glycosyl hydrolase, glucanase Crf1/allergen Asp F9, acid phosphatase PhoA, lysophospholipase Plb1, cell wall proteins Pst1, and other putative GPI proteins (Table 3). It appears that suppression of the GPI protein genes, especially the genes encoding Gel3 or cell wall proteins, might contribute to the cell wall defect of the mutant, though the function of Gel3 is still unknown.

**Table 2:** Chemical analysis of the cell wall.

Strain	Alkali soluble		Alkoli insoluble	
	glycoprotein (µg)	α-glucan (µg)	chitin (µg)	β-glucan (µg)
mutant	120±6	313±29	341±29	669±17
WT	138±9	321±19	343±24	741±19

Conidia were inoculated into 200 ml medium at a concentration of 10^6^ conidia/ml, and incubated at 37°C with shaking (200 rpm) for 36 h. The cell wall was extracted as described under Materials and Methods. Three aliquots of 10 mg lyophilized cell walls were used as independent samples for cell wall analysis and the experiment was repeated 3 times. The values (mean ± SD) shown are cell wall component per 10 mg dry cell walls.

**Table 3:** Differentially expressed GPI proteins encoding genes in the mutant.

Locus_tag	Description	Fold change
AFUA_4G03240	cell wall serine-threonine-richgalactomannoprotein Mp1	4.7
AFUA_2G01170	1,3-beta-glucanosyltransferase Gel1	11.8
AFUA_6G11390	1,3-beta-glucanosyltransferase Gel2	4.7
AFUA_6G00620	GPI anchored hypothetical protein	4.0
AFUA_3G01800	GPI anchored dioxygenase	9.3
AFUA_1G09650	GPI anchored protein	2.0
AFUA_7G00450	GPI anchored protein	3.6
AFUA_2G12850	1,3-beta-glucanosyltransferase Gel3	−43.4
AFUA_6G10290	GPI-anchored cell wall protein Pst1	−3.5
AFUA_3G01150	GPI anchored cell wall protein	−14.3
AFUA_8G04860	GPI anchored glycoprotein	−2.0
AFUA_1G17560	GPI anchored protein	−4.8
AFUA_6G10580	GPI anchored CFEM domain protein	−3.4
AFUA_1G10590	GPI anchored protein	−3.2
AFUA_4G02720	GPI anchored glycosyl hydrolase	−3.9
AFUA_4G03500	GPI anchored protein	−3.2
AFUA_2G01710	GPI anchored protein	−5.5
AFUA_6G02800	GPI anchored protein	−1.9
AFUA_8G04370	GPI anchored protein	−2.3
AFUA_1G05790	GPI anchored serine-rich protein	−1.5
AFUA_1G03570	acid phosphatase PhoA	−2.6
AFUA_1G16190	extracellular cell wall glucanase Crf1/allergenAsp F9	−1.8
AFUA_4G08720	lysophospholipase Plb1	−1.8

Microarray hybridization and analysis were carried out as described under Materials and Methods. The signal intensities were normalized using Feature Extraction Software (Agilent). Data were analyzed using Genespring Software 5.0. Genes with all signals present (flag = P) were selected for analysis. Genes were selected with P≤0.05, FC≥1.5 by T-test methods.

On the other hand, 7 GPI protein genes, including Gel1 and Gel2, were induced (Table 3). As Gel1 and Gel2 are GPI proteins responsible for cell wall synthesis in *A. fumigatus* [7], [8], the increase of Gel1 and Gel2 RNA levels might be triggered by the cell wall defect in the mutant through a compensatory mechanism.

In *A. fumigatus*, the compensatory mechanism for cell wall defects requires an activation of the cell wall integrity (CWI) signaling pathway, which consists of the mitogen-activated protein kinase (MAPK) cascade Bck1-Mkk2-MpkA/Slt2 and its upstream molecule PkcA/Pkc1 [33]. Microarray data showed that only *mpkA* (AFUA_4G13720) was induced 1.5-fold in this signaling pathway, while MAPKKK Bck1 (AFUA_3G11080) was suppressed 1.5-fold, and MAPKK Mkk2 (AFUA_1G05800) was not detected (Table 4). Real-time PCR analysis revealed that *mpkA* and *pkcA* (AFUA_5G11970) were induced 2.7- and 1.7-fold, respectively (Table 1). Using the anti-phospho-p44/42 MAPK (Erk1/2) antibody [33], we showed that calcofluor white, a cell wall disturbing compound that induces cell wall defects in fungi, induced activation of the MpkA in the WT, while MpkA was constitutively activated in the mutant (Fig.2B). These results suggest that, although MpkA was activated, the CWI signaling pathway (PkcA-Bck1-Mkk2-MpkA) was not activated in the mutant. The activation of MpkA might be triggered through other protein kinases instead of the Bck1-Mkk2. Although a number of genes encoding putative protein kinases were induced in the mutant, such as a protein kinase C substrate (AFUA_7G04110) and another upstream molecule of the MAPKKK, PakA (AFUA_2G04680) (Table 4), no evidence was obtained to show that these molecules served as upstream kinases to activate MpkA. Even though the CWI was not activated in the mutant, the activated MpkA may play an important role in the regulation of genes responsible for cell wall synthesis in the mutant.

**Table 4:** Protein kinases and sensors differentially expressed in the mutant.

Locus tag	Putative protein kinase	Fold change
AFUA_4G13720	MAP kinase MpkA	1.5
AFUA_1G12940	MAP kinase SakA	1.6
AFUA_3G05900	MAP kinase kinase Ste7	1.5
AFUA_1G15950	MAP kinase kinase (Pbs2)	1.5
AFUA_2G04680	sexual development serine/threonine kinase PakA	1.5
AFUA_7G04110	protein kinase C substrate	2.2
AFUA_6G14240	calcium sensor (NCS-1)	15.3
AFUA_3G09550	calcium/calmodulin dependent protein kinase	2.1
AFUA_1G04920	calmodulin-binding protein Sha1	1.5
AFUA_3G07050	WSC domain protein/Wsc2	1.5
AFUA_1G00530	thermoresistant gluconokinase family protein	7.4
AFUA_6G10240	sensor histidine kinase/response regulatorFos-1/TcsA	2.3
AFUA_8G06140	sensor histidine kinase/response regulator	2.0
AFUA_2G08470	GTP binding protein (Bud4)	1.6
AFUA_7G03720	serine/threonine protein kinase (Kin28)	1.9
AFUA_4G14740	serine/threonine protein kinase (Ark1)	6.8
AFUA_5G08570	cAMP-dependent protein kinase catalytic subunit	1.7
AFUA_1G06400	cAMP-dependent protein kinase-like	1.8
AFUA_5G04130	cyclin-dependent protein kinase PhoA	1.9
AFUA_4G06020	cyclin dependent kinase inhibitor Pho81	2.6
AFUA_5G08670	phosphoinositide 3-kinase	1.5
AFUA_1G11080	serine/threonine protein kinase Kin1	1.6
AFUA_2G07550	serine/threonine protein kinase (Ark1)	2.1
AFUA_5G05960	serine/threonine protein kinase	1.5
AFUA_5G14870	protein kinase	68.6
AFUA_5G06730	protein kinase	1.5
AFUA_6G08590	protein kinase	1.5
AFUA_2G00670	protein kinase	3.7
AFUA_2G09710	protein kinase (NpkA)	1.6
AFUA_4G08920	protein kinase	1.7
AFUA_3G03740	protein kinase	2.8
AFUA_5G09100	MAP kinase MpkC	−2.7
AFUA_3G11080	MAP kinase kinase kinase (Bck1)	−1.5
AFUA_5G08390	response regulator/Ssk1	−2.1
AFUA_3G10960	cell wall protein/MidA	−1.8
AFUA_5G08420	high osmolarity signaling protein Sho1	−1.6
AFUA_5G10020	sensor histidine kinase/response regulator	−35.0
AFUA_2G00660	sensor histidine kinase/response regulator TcsB/Sln1	−1.5
AFUA_4G02900	sensor histidine kinase/response regulator	−1.6
AFUA_3G07130	sensor histidine kinase/response regulator	−1.5
AFUA_5G08480	serine/threonine protein kinase	−1.8
AFUA_6G06720	serine/threonine protein kinase	−4.7
AFUA_3G01190	serine/threonine protein kinase	−2.1
AFUA_3G12670	serine/threonine protein kinase	−1.5
AFUA_2G09570	serine/threonine protein kinase	−2.6
AFUA_6G02300	serine/threonine protein kinase (Kcc4)	−36.0
AFUA_3G13990	cyclin-dependent protein kinase Ssn3	−2.0
AFUA_5G05510	cyclin-dependent protein kinase Sgv1	−1.6
AFUA_5G12660	phosphotidylinositol kinase Tel1	−1.6
AFUA_1G10980	sphingosine kinase (SphK)	−3.6
AFUA_7G03760	phosphatidylinositol 4-kinase (STT4)	−2.4
AFUA_6G03255	protein kinase	−17.6
AFUA_8G05980	protein kinase	−1.6
AFUA_3G02500	protein kinase	−12.5
AFUA_5G05750	protein kinase	−1.5
AFUA_7G03750	cell-cycle checkpoint protein kinase	−1.5

Microarray hybridization and analysis were carried out as described under Materials and Methods. The signal intensities were normalized using Feature Extraction Software (Agilent). Data were analyzed using Genespring Software 5.0. Genes with all signals present (flag = P) were selected for analysis. Genes were selected with P≤0.05, FC≥1.5 by T-test methods.

Recently, Wsc1, Wsc2, Wsc3, and MidA have been identified as cell surface stress sensors in *A. fumigatus* [34]. The function of Wsc2 is still unknown. Wsc1, Wsc3, and MidA were shown to be responsible for detecting cell wall defects and activating the CWI pathway to modulate growth and cell wall remodeling [34]. In our study, only the *wsc2* gene (AFUA_3G07050) was induced 1.5-fold, while the *midA* gene (AFUA_3G10960) was suppressed 1.8-fold in the mutant. Additionally, the nutrient and osmolarity sensor molecules Sho1 and Sln1 were suppressed in the mutant, while Rho GTPase ModA, MAPKK Pbs2 and MAPK SakA/Hog1 were induced, which clearly suggests an occurrence of other stress conditions (Table 1 and 4). In addition to Wsc2, there are probably several other induced stress sensors or response regulators which may respond to the stress in the mutant (Table 4). Taken together, it is likely that instead of the cell wall defect, a yet unknown stress signal made a major contribution to the activation of MAPK in the mutant.

### Phosphatidylinositol (PtdIns) metabolism and PtdIns signaling in the mutant

**Figure 3:**
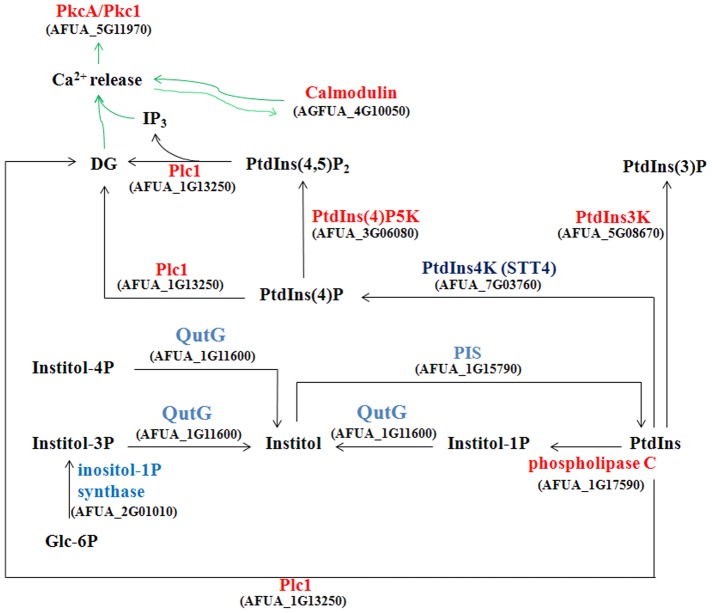
Phosphatidylinositol (PtdIns) metabolism and signaling in the mutant. As revealed by transcriptome data, PtdIns synthesis was suppressed in the mutant, including QutG (inositol monophosphatase), myo-inositol-1-phosphate synthase, and PIS (CDP-diacylglycerol-inositol 3-phosphatidyltransferase). On the other hand, the genes encoding PtdIns4(P)5K (PtdIns-4-phosphate 5-kinase), PtdIns3K (PtdIns 3-kinase), Plc1 (PtdIns-4,5-bisphosphate phosphodiesterase) and calmodulin were induced at least 1.5-fold. PtdIns3K catalyzes the conversion of PtdIns into PtdIns(3)P (PtdIns-3-phosphate), whereas PtdIns4(P)5K catalyzes the conversion of PtdIns(4)P (PtdIns-4-phosphate) into PtdIns(4,5)P_2_ (PtdIns-4,5-biphosphate). PtdIns, PtdIns(4)P and PtdIns(4,5)P_2_ all can be hydrolyzed into diacylglycerol (DG) by Plc1. IP_3_ diffuses in the cytoplasm and binds to its receptor (IP3R), which is an intracellular ligand-gated Ca^2+^ release channel localized primarily in the endoplasmic reticulum (ER). Then Ca^2+^ in the ER would be released into the cytoplasm. The Ca^2+^ can activate PKC (protein kinase C) and calmodulin which regulates the Ca^2+^ release channel in return. DG also can induce the Ca^2+^ release and activates the PKC PkcA/Pkc1. Red, induced protein; blue, suppressed protein. Green arrow indicates activation.

As the *afpig-a* gene encodes the enzyme responsible for the transfer of GlcNAc to PtdIns on the cytoplasmic face of the ER, it is expected that suppression of the *afpig-a* gene may affect PtdIns metabolism. Transcriptome analysis revealed that the PtdIns synthesis pathway was suppressed (Fig.3), including inositol monophosphatase QutG (AFUA_1G11600), myo-inositol-1-phosphate synthase (AFUA_2G01010), and CDP-diacylglycerol-inositol 3-phosphatidyltransferase PIS (AFUA_1G15790). The gene encoding QutG was confirmed to be repressed 5.7-fold by RT-PCR (Table 1). On the other hand, the genes encoding PtdIns-4-phosphate 5-kinase (PtdIns(4)P5K) (AFUA_3G06080), PtdIns 3-kinase (PtdIns3K) (AFUA_5G08670), PtdIns-4,5-bisphosphate phosphodiesterase (PLC) Plc1 (AFUA_1G13250) and calmodulin (AFUA_4G10050) were induced at least 1.5-fold (Fig.3), among which PtdIns3K, Plc1, and calmodulin were confirmed to be induced about 2-fold by RT-PCR (Table 1). PtdIns3K catalyzes the conversion of PtdIns into PtdIns-3-phosphate (PtdIns(3)P), whereas PtdIns4(P)5K catalyzes the conversion of PtdIns-4-phosphate (PtdIns(4)P) into PtdIns-4,5-biphosphate (PtdIns(4,5)P_2_). Both PtdIns and PtdIns(4,5)P_2_ can be hydrolyzed into diacylglycerol (DG) by PLC. DG and IP_3_ function as second messenger signals to stimulate the release of ER lumenal calcium into the cytosol. Therefore, the induced PtdIns3K, PtdIns4(P)5K, Plc1, and calmodulin suggest a stimulation of the PtdIns signaling pathway and release of Ca^2+^ in the mutant. As DG and IP_3_ are physiological activators of Pkc1, it is likely that PkcA/Pkc1 and its downstream molecule MpkA were activated by the PtdIns signaling in the mutant. In contrast to PtdIns3K and PtdIns(4)P5K, PtdIns4K/Stt4 (AFUA_7G03760) was suppressed, which may lead to a decrease of PtdIns(3,4)P_2_ in the mutant.

### Enhanced protein synthesis and endoplasmic reticulum (ER) stress in the mutant

Most of the differentially expressed genes responsible for amino acid metabolism, aminoacyl-tRNA biosynthesis, and ribosome were up-regulated in the mutant at least 2.0-fold (Table S4 and Fig. S1), suggesting that suppression of GPI synthesis induces enhanced protein synthesis. The genes encoding ribosome subunit L22e and S6e were confirmed to be induced 2.5- and 3.2-fold, respectively (Table 1). Meanwhile, the genes contributing to proteasome and ubiquitin-mediated proteolysis were also induced (Table S4). Among them, the genes encoding E2 ubiquitin-conjugating enzyme subunit UBE2G1 and ubiquitin ligase complex F-box subunit GRR1 were confirmed to be induced 2.0- and 2.1-fold, respectively (Table 1). Additionally, genes encoding Hsp70 family proteins were induced at least 1.8-fold, including chaperone Lhs1/Orp150, HscA/SSB, BiP/Kar2, and two other Hsp70 family proteins (AFUA_3G13740 and AFUA_7G08575) (Table 5).

**Table 5:** Necroptosis related genes in the mutant.

Protein required for necroptosis	Locus tag in *A. fumigatus* genome	Protein in *A. fumigatus*	Fold change
PYGL	AFUA_1G12920	glycogen phosphorylase GlpV/Gph1	3.3
GLUL	AFUA_6G03530	glutamine synthetase	1.5
GLUD1	AFUA_2G06000	NAD^+^ dependent glutamate dehydrogenase	2.2
Hsp70 family	AFUA_1G15050	Hsp70 family chaperone Lhs1/Orp150	2.1
	AFUA_8G03930	Hsp70 chaperone (HscA)/heat shock protein SSB/splicesome	2.7
	AFUA_2G04620	Hsp70 chaperone BiP/Kar2	2.0
	AFUA_2G02320	Hsp70 chaperone (BiP)	1.8
	AFUA_3G13740	HSP70 family protein	2.3
	AFUA_7G08575	Hsp70 family chaperone	172
JNK1	AFUA_1G12940	MAP kinase SakA	1.6
AIF	AFUA_7G02070	AIF-like mitochondrial oxidoreductase Nfrl	7.1
Cyclophilin familyCYPD	AFUA_8G03890	Peptidyl-prolyl cis-trans isomerase H	2.0
	AFUA_3G07430	peptidyl-prolyl cis-trans isomerase/cyclophilin	2.8
	AFUA_1G01750	peptidyl-prolyl cis-trans isomerase	1.5
	AFUA_6G02140	peptidyl prolyl cis-trans isomerase (CypC)	2.3
PKA	AFUA_5G08570	cAMP-dependent protein kinase catalytic subunit, putative	1.7
	AFUA_1G06400	cAMP-dependent protein kinase-like	1.8
Glyoxalase	AFUA_7G05015	glyoxalase family protein	7.1
	AFUA_7G05010	glyoxalase family protein	2.8
	AFUA_3G06020	glyoxalase family protein	4.9
Rab7/Ypt7	AFUA_5G12130	Rab small monomeric GTPase Rab7	1.6
SMase	AFUA_2G01600	sphingomyelin phosphodiesterase	−2.1
ceramidase	AFUA_4G12330	alkaline dihydroceramidase Ydc1	−1.6
PARP1	AFUA_5G07320	poly(ADP)-ribose polymerase PARP	−2.1
calpains	AFUA_6G07970	Calpain-like protein	−10.2

Microarray hybridization and analysis were carried out as described under Materials and Methods. The signal intensities were normalized using Feature Extraction Software (Agilent). Data were analyzed using Genespring Software 5.0. Genes with all signals present (flag = P) were selected for analysis. Genes were selected with P≤0.05, FC≥1.5 by T-test methods.

ER stress is known to induce an over-expression of Hsp70 family protein Lhs1/Orp150, ubiquitin protein subunit Bre1 and proteasome subunit Rpt6 in a mutant deficient in N-glycan processing [35]. As shown in Fig.2C, Western blotting analysis showed that the amount of Gel1 protein was increased in the cytosol and decreased in the membrane of the mutant, suggesting that up-regulation of the *gel1* gene did not completely restore the membrane bound Gel1 to a level similar to the WT and led to an accumulation of the Gel1 inside the mutant cells. In contrast, the membrane bound Gel4 and Ecm33, which were not found to be differentially expressed in the mutant, were similar to those in the WT. Therefore, we propose that the enhanced protein synthesis and accumulation of the GPI protein precursors in the ER led to ER stress in the mutant.

**Figure 4:**
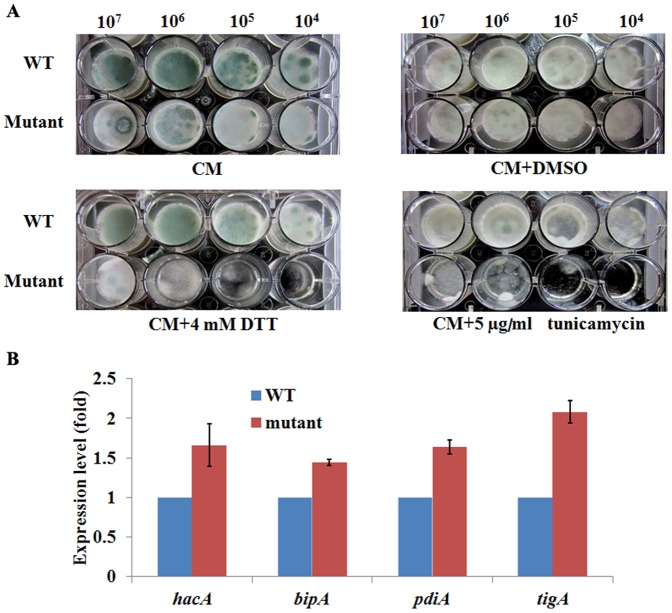
Evidence for ER stress in the conditional *afpig-a* mutant. In A, a series of 10-fold dilutions (10^7^–10^4^ cells) of spores were inoculated into 1 ml liquid CM containing 1‰ DMSO, 4 mM DTT or 5 µg/ml tunicamycin (Sigma) and incubated at 37°C for 36 h. The experiment was repeated 3 times; In B, detection of UPR related genes by Real-time PCR. Results are presented as mean ± SD;

When the mutant was treated with ER stress inducing agents such as DTT and tunicamycin, an increased sensitivity to DTT or tunicamycin was observed (Fig.4A). These results confirmed that repression of the *afpig-a* gene resulted in ER stress in the mutant. ER stress has been shown to activate the unfolded protein response (UPR) in *A. fumigatus*. The *A. fumigatus ΔhacA* mutant is unable to activate the UPR in response to ER stress and hypersensitive to agents that disrupt ER homeostasis or the cell wall [36]. In our mutant, the *hacA*, *bipA*, *pdiA* and *tigA* genes were found to be induced (p<0.05) (Fig.4B), suggesting an activation of the UPR signaling by ER stress.

### Increased cytosolic Ca^2+^ in the mutant

**Figure 5:**
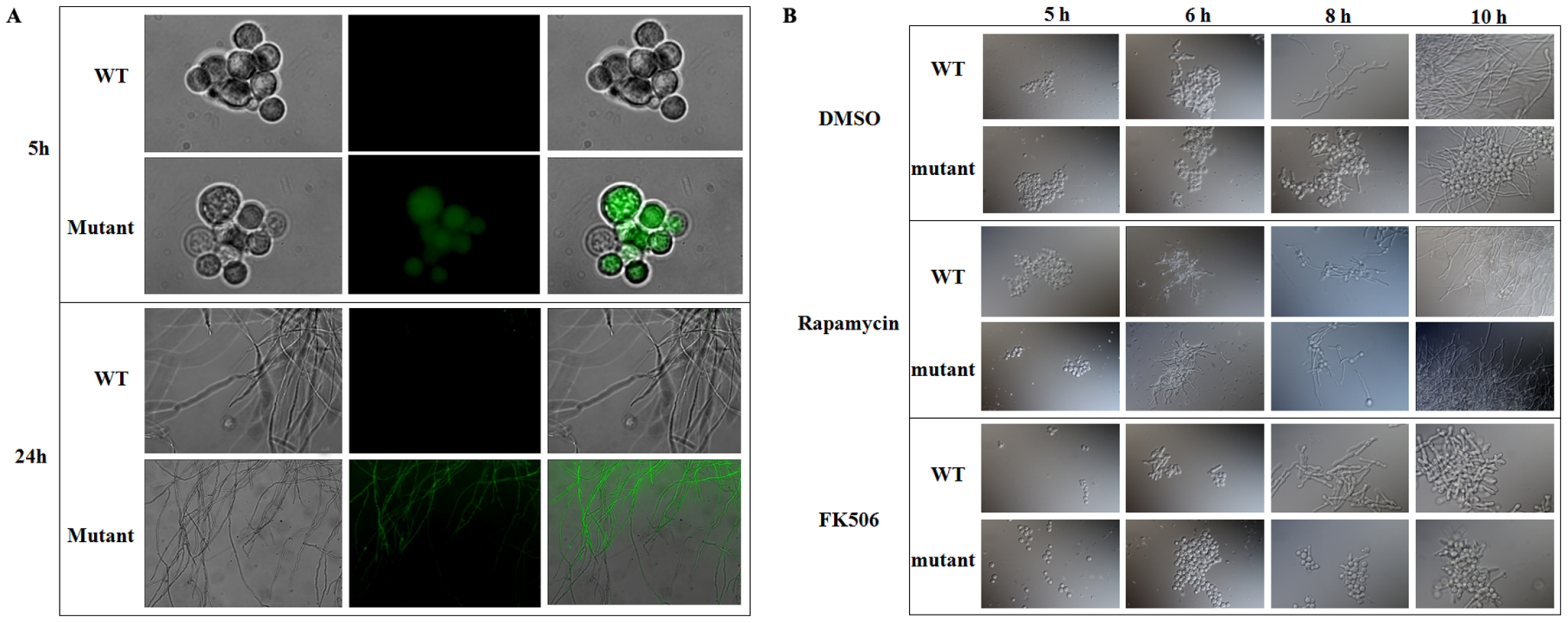
Evidence for increased cytosolic Ca^2+^ in the conditional *afpig-a* mutant. In A, 2×10^8^ spores were inoculated into 200 ml CM media and cultivated at 37°C, 200 rpm for 5 h or 24 h. Spores or mycelia were stained with Fluo 3-AM and examined under the fluorescene microscope (Zeiss); in B, spores were inoculated into liquid CM containing 1cene micr µM FK506 or 10 nM rapamycin at a concentration of 10^6^ conidia/ml and incubated at 37°C with shaking (200 rpm) for different amounts of time, mycelia were detected under a differential interference contrast (DIC) microscope (Olympus).

In addition to PtdIns signaling, ER stress is also known to induce the release of ER luminal Ca^2+^ into the cytosol [37]. It is not surprising to find that calmodulin, calcium sensor NCS-1, calcium/calmodulin dependent kinase, and calmodulin-binding protein Sha1 were induced in the mutant (Table 1 and 4). These observations strongly suggest an activation of Ca^2+^ signaling. To gain direct evidence, we detected the cytosolic Ca^2+^ of the mutant with Fluo 3-AM. Our results showed that only mutant cells were positively stained by Fluo 3-AM (Fig.5A), indicating a remarkable increase of the cytosolic Ca^2+^. We also observed that retarded growth of the mutant under repressive conditions was enhanced by FK506 and restored by rapamycin (Fig.5B). As FK506 and rapamycin are known to enhance and reduce Ca^2+^ signaling respectively [38]–[43], these observations also confirm an increase of cytosolic Ca^2+^ in the mutant.

### Autophagic processes in the mutant

**Figure 6:**
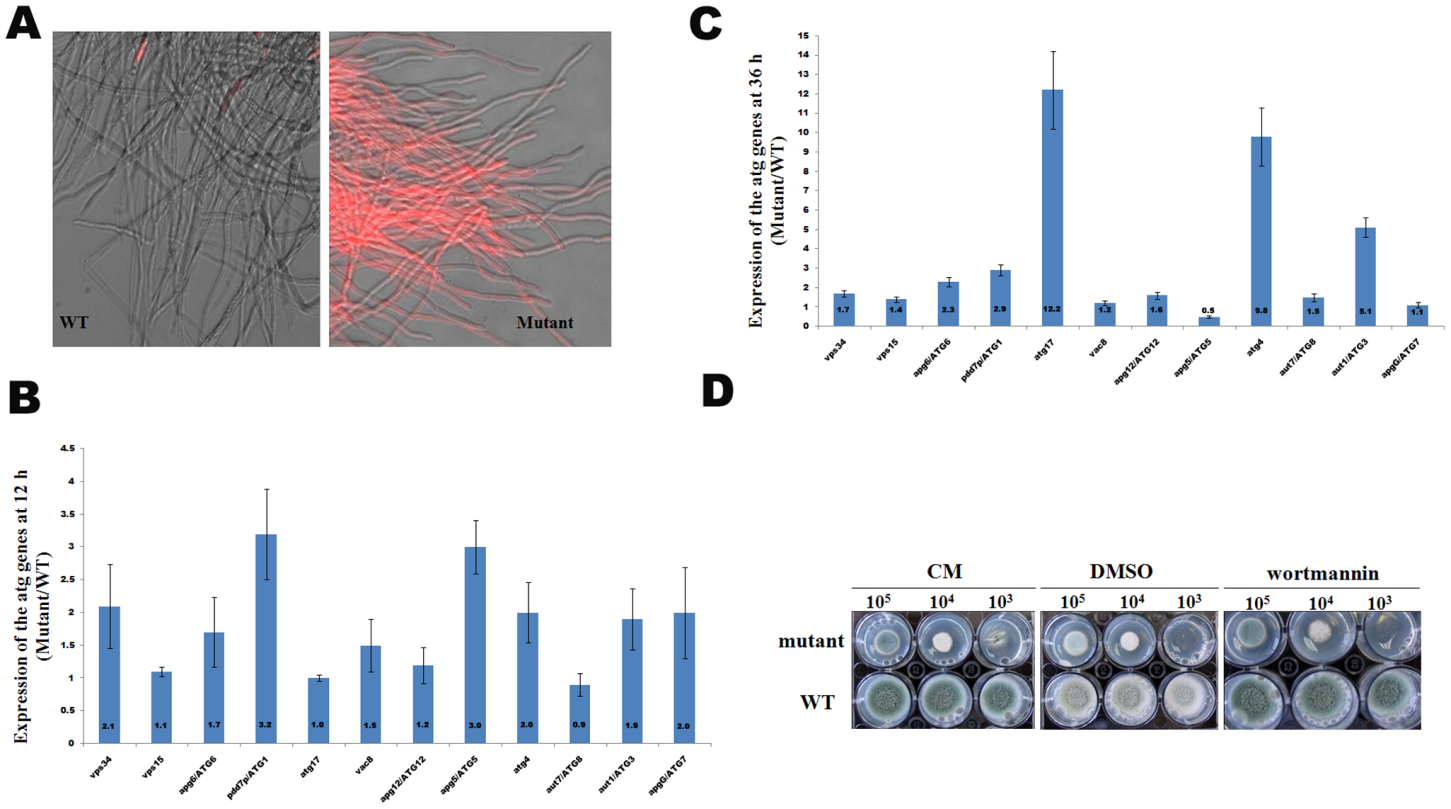
Evidence for autophagic processes in the conditional *afpig-a* mutant. In A, after growing in liquid CM at 37°C for 24 h, the mycelia were collected, stained with propidium iodide (PI), and then examined under the fluorescene microscope (Zeiss); in B and C, strains grown in CM at 37°C for 12 h or 36 h were examined by Real-time PCR. Results are presented as mean ± SD; in D, a series of 10-fold dilutions (10^3^–10^5^ cells) of spores was spotted onto solid CM containing 1‰ DMSO, or 0.2 µM wortmannin. The plates were incubated at 37°C for 36 h and photographed.

Among the phenotypes displayed by the conditional expression mutant, the most significant pheneotype was increased cell death (Fig.6A). Propidium iodide (PI) staining revealed that about 45.5% of the mutant mycelia were PI positive, while only around 21.43% of the WT mycelia were PI positive.

Three different types of programmed cell death (PCD) have been defined, including apoptosis, autophagy (autophagic cell death), and necroptosis [44], [45]. Apoptosis is a caspase-dependent PCD characterized by elevated caspase activity, loss of membrane asymmetry and DNA fragmentation. Metacaspase Yca1p is the only caspase in *S.cerevisiae*. It has been reported that ER stress is one of the factors that trigger an Yca1p-dependent apoptotic process in *S.cerevisiae* [46]. Similarly, the *A. fumigatus* metacaspases CasA and CasB are found to contribute to the apoptotic-like loss of membrane phospholipid asymmetry in the stationary phase and facilitate growth under ER stress [47]. In our study, the *casA* gene (AFUA_1G06700) was suppressed in the mutant. Biochemical assays revealed that caspase-1, caspase-3, and caspase-8 activity in the mutant were reduced to 45.9±2.6%, 75.3±7.5%, and 75.9±1.7%, respectively, as compared with the WT. The percentage of phosphatidylserine externalization in the mutant (12.7±5.2%) was similar to that (13.4±3.873%) in the WT and no DNA fragmentation was detected. These results clearly demonstrated that increased cell death of the mutant was not mediated by caspase-dependent apoptosis.

In contrast to *casA* suppression, several autophagy-related genes were induced in the mutant, such as the *apg12*/*ATG12* (AFUA_6G09165), *aut1*/*ATG3* (AFUA_5G08170), *apg9*/*ATG9* (AFUA_6G12350), *vac8* (AFUA_5G13540), and *rab7* (AFUA_5G12130) (Table S2 and Table 5). In *S. cerevisiae*, the formation of the autophagosome is initiated by recruiting the Atg1-Atg13-Atg17 complex (Atg1 complex) at the phagophore assembly site (PAS), the organizing site on the vacuole for phagophore formation. During autophagosome formation, both the Atg12-Atg5-Atg16 complex (Atg12 complex) and the Atg8-PE (phosphatidylethanolamine) conjugate are localized at the PAS and drive membrane expansion and vesicle completion. Atg9 is thought to act as a ‘membrane carrier’ during the phagophore assembly process. Vac8 is a component of the Atg1 complex and Atg3 is an E2-like enzyme responsible for conjugating the Atg8/LC3 to PE [48], [49]. The Rab7/Ypt7 is required for the fusion of the autophagosome with the lysosome/vacuole in yeast and mammalian cells [37]. Up-regulation of the *vac8*, *apg12*, and *aut1* genes was confirmed (Table 1).

Among the *S.cerevisiae* Atg proteins, four subgroups are thought to be the core molecular machinery essential for autophagosome formation, which includes the PtdIns3K/Vps34 complex I, the Atg1 complex, the ubiquitin-like protein Atg12 and Atg8 conjugation systems, and two transmembrane proteins Atg9/mAtg and VMP1 [49]. Among these proteins, PtdIns3K/Vps34 plays an important role in phagosome formation either by directly forming the PtdIns3K/Vps34 complex I or indirectly recruiting several Atg proteins at the PAS through its kinase activity product PtdIns(3)P. As the PtdIns3K and several Atg proteins were induced in the mutant, we therefore further analyzed the genes belonging to these four subgroups by Real-time PCR. When the mutant was cultivated in CM at 37°C for 12 h, *Vps34* and *apg6*/*ATG6*/*VPS30*, which encode components of the PtdIns3K/Vsp34 complex I, were induced at least 1.7-fold; the *pdd7*/*ATG1* and *vac8* genes, which encode the key components of the Atg1 complex and act as down-stream targets of the TORC1, were induced 3.2- and 1.5-fold respectively; and apg*12*/*ATG12*, *apg5*/*ATG5*, *atg4*, and *aut1*/*ATG3*, which encode proteins belonging to the ubiquitin-like protein conjugation systems, were induced on a range of 1.2 to 3.0 fold (Fig.6B). The *apgG*/*APG11*, a gene encoding the Atg11 that links peroxisome destined for degradation to the PAS [37], was also induced. Prolonged incubation of the mutant for 36 h led to an induction of the *vps34*, *vps15*, *apg6*/*ATG6*, *pdd7*/*ATG1*, *atg17*, *vac8*, *apg12*/*ATG12*, *atg4*, *aut7*/*ATG8*, and *aut1*/*ATG3* genes (Fig.6C).

**Figure 7:**
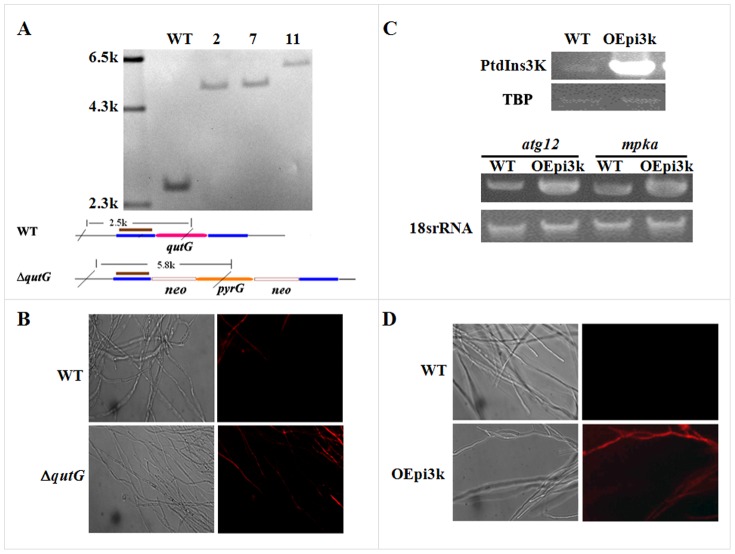
Constructions of the QutG deletion mutant and PtdIns3K over- expression strain in *A. fumigatus*. The null mutant *ΔqutG* (A) and PtdIns3K overexpression strain OEpi3k (C) were constructed and confirmed as described under Materials and Methods. After growing in liquid CM at 37°C for 12 h (D) or 24 h (B), the mycelia were stained with propidium iodide (PI) and examined under the fluorescene microscope (Zeiss).

As transcriptome analysis revealed that suppression of GPI synthesis led to the inhibition of PtdIns synthesis and the activation of PtdIns signaling in the mutant. We therefore propose that PtdIns signaling was one of the key factors to induce autophagy in the mutant. To verify our hypothesis, we deleted the gene encoding QutG to generate the *ΔqutG* strain and over-expressed PtdIns3K to generate the PtdIns3K overexpression strain OEpi3k. It turns out that both mutants displayed an increased incidence of cell death (Fig.7), indicating a key role of the activation of PtdIns signaling in inducing autophagy in the mutant. On the other hand, wortmannin, an autophagy inhibitor that specifically inhibits PtdIns3K, did not affect growth and conidiation of the mutant (Fig.6D), suggesting the existence of another mechanism for the cell death in the mutant.

### Morphological evidences of programmed cell death in the mutant

**Figure 8:**
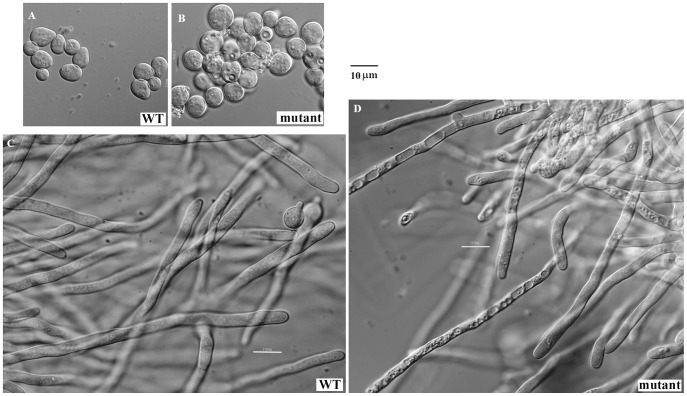
Differential interference contrast microscopy (DIC) of the mutant. 2×10^8^ conidia were inoculated into 200 ml liquid CM media and incubated at 37°C for 5 h (A and B) or 11 h (C and D) with shaking (200 rpm). The conidia and mycelia were collected and examined under the differential interference contrast microscope (DIC) (Olympus). The picture shows typical conidia (B) and mycelium (D) of the mutant strain.

Morphologically, autophagy is accompanied by massive vacuolization of the cytoplasm, while necroptosis is characterized by a gain in cell volume, swelling of organelles, plasma membrane rupture and subsequent loss of intracellular contents [45]. The averge diameter of the mutant conidia was 2.2±0.2 µm, while that of the WT was 1.6±0.2 µm. Under the DIC microscope, multivacuolar vesicles and massive vacuolization were observed in about 30% of the germinating mutant conidia grown in CM at 37°C for 5 h (Fig.8B) and the mutant mycelia grown in CM at 37°C for 11 h (Fig.8D), while such phenotype only existed in 7.5% of the WT cells. Both multivacuolar vesicles and massive vacuolization at an early stage of germination may suggest an occurrence of autophagy and possible necroptosis in the mutant.

**Figure 9:**
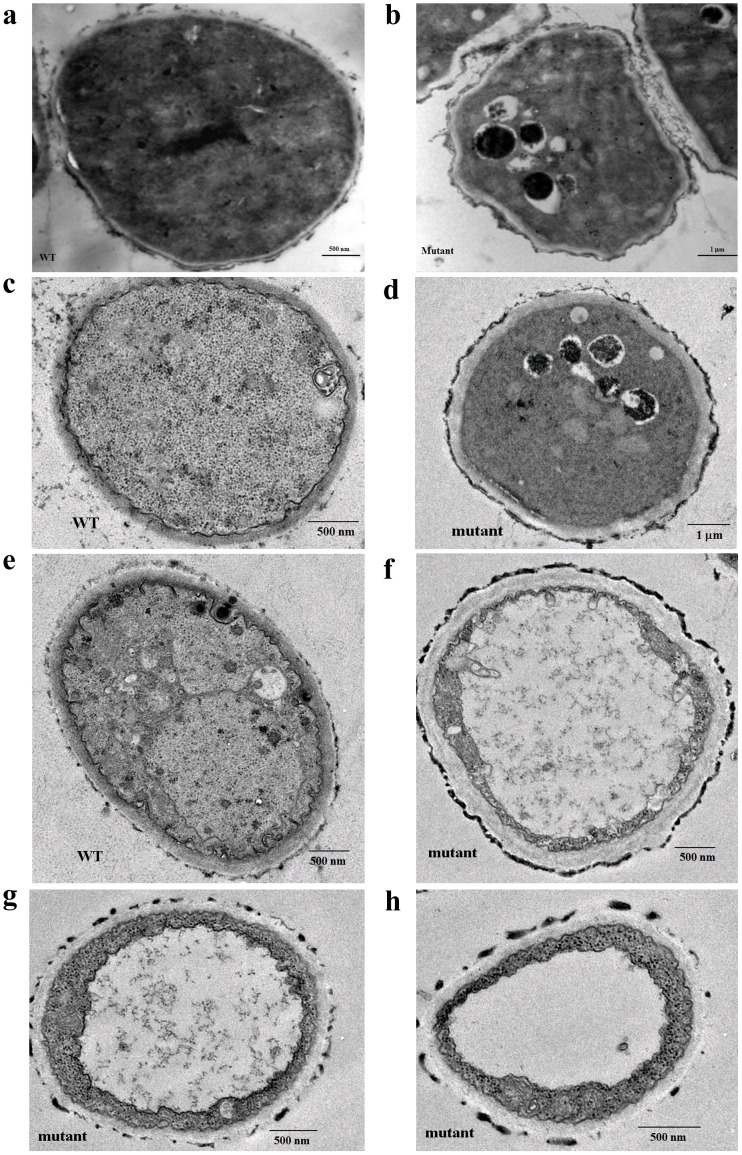
Transmission electron microscopy (TEM) of the mutant. 2×10^8^ conidia were inoculated into 200 ml liquid CM and incubated at 37°C with shaking (200 rpm). After incubation for 7 h (a-b), 12 h (c-d), or 24 h (e-h), samples were prepared as described under Materials and Methods and examined under the transmission electron microscope (TEM) (FEI).

**Figure 10:**
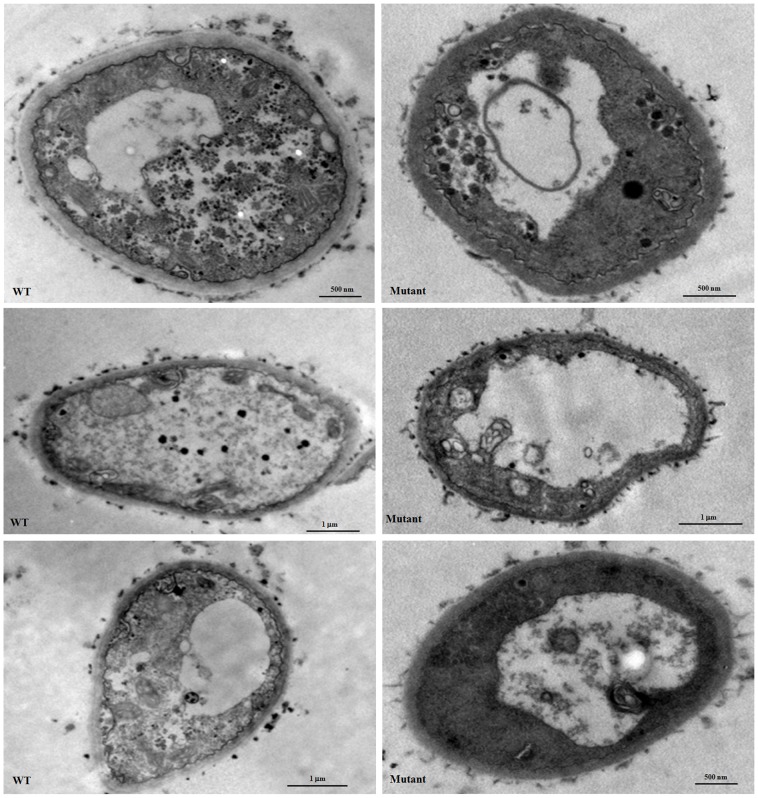
Transmission electron microscopy (TEM) of the mutant. 2×10^8^ conidia were inoculated into 200 ml liquid CM and incubated at 37°C with shaking (200 rpm). After incubation for 36 h, samples were prepared as described under Materials and Methods and examined under the transmission electron microscope (TEM) (FEI).

Under the transmission electron microscope (TEM), autophagosomes were observed in the mutant strain grown in CM for 7 h or 12 h (Fig.9, b and d), as the formation of the autophagosome is a central process of autophagy, our results clearly demonstrated an occurrence of the autophagic process in the mutant. In addition to autophagosome, both massive vacuolization and translucent cytoplasma were found in the mutant grown in CM for 24 h (Fig.9, f–h) or 36 h (Fig.10). After growing in CM for 36 h, 47.1±9.1% of the cell volume of the mutant strain was filled with vacuolar or translucent vesicles, while only 15.7±5.1% of the WT cells was occupied by vesicles. Furthermore, a remarkable release of the GPI proteins Ecm33, Gel1, and Gel4 into culture supernatant implied a disintegration of plasma membrane in the mutant (Fig.2C). These observations demonstrated an increased necroptosis in the mutant.

Over the past two decades, a number of molecules and processes have been characterized as initiators, modulators or effectors of necroptosis [45], [50], [51]. A genome wide siRNA screen revealed that proteins involved in necroptosis are especially abundant in the processes of glutathione metabolism, GPI-anchor biosynthesis, ribosomal proteins and translation [50]–[52]. Transcriptome analysis of the conditional expression mutant revealed that some of the molecules required for necroptosis were induced at least 1.5-fold, such as mitochondrial ADP/ATP carrier protein Nfr1/AIF, cyclophilins, SakA/Hog1/JNK1, Hsp70 family proteins, cAMP-dependent protein kinase PKA, glyoxalase family proteins, and GTPase Ypt7/Rab7 (Table 5). Additionally, the differentially expressed genes in the mutant were enriched in several pathways or processes involved in necroptosis, such as protein synthesis (Table S4), glycogenolysis and glycolysis (Fig.S2), glutaminolysis (Table 5), TCA cycle (Fig.S3), oxidative phosphorylation (Fig.S4), peroxidation (Fig.S5), and advanced glycation end products (AGEs) (Table 5). Although several other proteins required for necroptosis were suppressed, such as sphingomyelin phosphodiesterase (SMase), ceramidase Ydc1, poly(ADP)-ribose polymerase (PARP), and calpains (Table 5), most molecules and processes essential for necroptosis were found induced in the mutant and some of them were confirmed by Real-time PCR (Table 1). Therefore, we propose that the increased cell death of the mutant might also be mediated by necroptosis.

In mammalian cells, when caspase activity is inhibited, the receptor-interacting protein 1 (RIP1) and RIP3 are activated and form a multiprotein complex containing RIP1 and RIP3, called necrosome, which stimulates necroptosis. The RIP homologs have not been identified yet in *S.cerevisiae* or *A. fumigatus.* In an attempt to identify the necroptotic regulator in *A. fumigatus*, we tried to detect the *A. fumigatus* RIP1 and RIP3 homologs with commercially available RIP1 and RIP3 antibodies. As a result, only one protein band was detected by the RIP3 antibody, and no protein was detected by the RIP1 antibody. The protein band detected in the mutant by the RIP3 antibody was stronger than that in the WT. Immuno-precipitation followed by LC-MS/MS revealed that one of the potential targets of the RIP3 antibody was a putative protein kinase (AFUA_6G02590). However, this putative protein kinase only contains a kinase domain, while the homotypic interaction motif (RHIM) that is required for interaction with the RIP1 is absent [53], [54]. As the assembly of the RIP1-RIP3 complex can be inhibited by necrostatin-1, a small molecule that allosterically blocks the kinase activity of RIP1 [35], [44], [50], we further tested the effect of necrostatin-1 on the mutant. It turned out that nercostatin-1 did not suppress the death of the mutant. Based on these results, it is likely that the necroptotic process in *A. fumigatus* is RIP-independent and that either the mechanism or the RIP3-like protein are different from those in mammalian cells.

## Discussion

In this study, a conditional expression *afpig-a* mutant was constructed and displayed phenotypes similar to those of the null mutant [13]. Under suppressive conditions, expression of the *afpig-a* gene was suppressed by 54% in the mutant. As some GPI proteins are required for cell wall synthesis, it is conceivable that reduced expression of the *afpig-a* gene led to insufficient GPI anchor synthesis, failure of GPI protein trafficking to the plasma membrane, and eventually cell wall defects. In *A. fumigatus*, the MAPK MpkA/Slt2 plays a major role in cell wall integrity [55]. In this study, we found that the *mpkA* gene was induced and the MpkA protein was constitutively phosphorylated in the mutant (Table 1 and Fig.2B). However, Bck1 and Mkk2, the upstream molecules in the MAPK cascade Bck1-Mkk2-MpkA/Slt2, were suppressed, suggesting that MpkA is activated through pathways other than the CWI pathway. Indeed, an increased expression of the *mpkA* gene was observed in strain OEpi3k that over-expressed PtdIns3K (Fig.7), which suggests that activation of PtdIns signaling may be one of the causes leading to MpkA activation through its upstream molecule PkcA/Pkc1 in the conditional expression mutant. Still, it is likely that activation of MpkA would up-regulate the enzymes responsible for cell wall synthesis, such as Gel1, Gel2 and chitin synthase (AFUA_2G13430).

As the *afpig-a* gene encodes the enzyme responsible for the transfer of GlcNAc to PtdIns, it is expected that suppression of the *afpig-a* gene may affect PtdIns metabolism in the mutant. Transcriptome analysis revealed that the PtdIns synthesis was inhibited and conversion of PtdIns to PtdIns(3)P, DG and IP_3_ was induced in the mutant (Fig.3). On the other hand, insufficient GPI anchor supply may lead to the accumulation of un-anchored proteins in the ER and thus ER stress. Several pieces of evidence have been obtained to show the occurrence of ER stress in the mutant: (i) an over-expression of Hsp70 family proteins such as Lhs1/Orp150, HscA, and BiP/Kar2 was detected in the mutant (Table 5), suggesting an accumulation of protein in the ER; (ii) a number of genes encoding proteins required for the proteasome and ubiquitin-mediated proteolysis were induced, suggesting an induction of ER-mediated degradation (ERAD) in the mutant; (iii) the mutant was more sensitive to ER stress inducing agents; and (iv) expression of *hacA*, *bipA*, *pdiA* and *tigA* were induced, suggesting an activation of UPR in the mutant.

Both activation of PtdIns signaling and ER stress are known to stimulate release of the ER lumenal Ca^2+^. Indeed, we did obtain direct evidence to show a remarkable increase of cytosolic Ca^2+^ in the mutant (Fig.5A). Therefore, it is reasonable to conclude that increased Ca^2+^ efflux from the ER in the mutant was the result of both activation of the PtdIns pathway and ER stress.

Results obtained in this study show that the mutant died through autophagy instead of apoptosis. In *S. cerevisiae*, both the Vps34 protein and its kinase activity are essential for autophagy. Vps34 is a key component of the PtdIns3K complex I that is required for the induction of autophagy and its PtdIns3K kinase activity is essential for generating PtdIns(3)P at the PAS to recruit Atg proteins [37], [48]. Transcriptome analysis revealed that suppression of GPI synthesis led to the inhibition of PtdIns synthesis and the activation of PtdIns signaling in the mutant, which were featured with suppression of the gene encoding QutG and induced PtdIns3K. Our experiments also confirmed that both *ΔqutG* mutant and PtdIns3K overexpression strain displayed an increased incidence of cell death (Fig.7). These results clearly demonstrated that the autophagy that occurred in the mutant was induced by the inhibition of PtdIns synthesis, as well as the induction of PtdIns3K.

On the other hand, an increasing number of studies indicate that autophagy is induced by ER stress in organisms ranging from yeast to mammals. In *S. cerevisiae*, ER stress induces autophagy through the UPR signaling pathway, in which the Hsp70 family protein Grp78/BiP may play an important role. In mammalian cells, ER stress induced the release of lumenal Ca^2+^ into the cytosol activating calcium-activated calmodulin-dependent kinase kinase-β (CaMKKβ), AMPK and further inducing autophagy [37]. In our study, we have shown that ER stress and thus UPR signaling were induced in the mutant. Based on our results, it appears that autophagic processes were induced by both PtdIns signaling and UPR signaling. The observation that wortmannin did not affect growth of the mutant (Fig.6D) indirectly supports our conclusion. One plausible explanation for this is that the autophagic death of the mutant was still induced by UPR signaling when PtdIns3K was inhibited by wortmannin. Another possibility is that the mutant died through necroptosis when autophagy was inhibited by wortmannin as increased necroptosis was observed in the mutant.

Although autophagy has been proposed to be one of several execution mechanisms for necroptosis as autophagy vesicles are commonly observed in necroptotic cells [44], [56], others have argued that autophagy inhibits necroptosis [52]. In our study, both massive vacuolization and induction of Atg genes were observed in the mutant cultivated for 11–12 h (Fig.8D and Fig.6B), indicating an occurrence of both necroptosis and autophagy at the early stages of growth. Under the TEM, autophagosomes were seen in the mutant grown in CM for 7 h and 12 h (Fig.9, b and d), while massive vacuolization and translucent cytoplasma were found in the mutant grown in CM for 24 h (Fig.9, f-h) and 36 h (Fig.10). These observations suggest that both autophagy and necroptosis were induced in the mutant.

In mammalian cells, RIP1 serine/threonine kinase activity is essential for necrotic death but dispensable for apoptosis. Activation of caspase-8 activity inactivates RIP1 and RIP3 by proteolytic cleavage and triggers caspase-dependent apoptosis. If caspase-8 activity is inhibited, RIP1 and RIP3 are phosphorylated and form the necrosome, upon which necroptosis is switched on [35], [44], [45], [50], [54], [57]–[64]. In our study, suppression of *casA* and reduced caspase activities were detected in the mutant. However, we failed to identify an analog of RIP1 in *A. fumigatus*. The observation that necrostatin-1 did not prevent necroptotic cell death in the mutant also suggests the absence of a RIP-1 analog in *A. fumigatus* (Fig.S6B). Meanwhile, absence of the RHIM domain, which is required for interaction with RIP1, in the protein kinase (AFUA_6G02590) co-precipitated with the RIP3 antibody suggests that *A. fumigatus* might possess a RIP1-independent and RIP3-dependent mechanism that is different from mammalian cells. To verify this hypothesis, further experiments need to be carried out.

In conclusion, suppression of GPI anchor synthesis led to ER stress and activation of PtdIns signaling in *A. fumigatus*. Although the mechanism is not completely understood, it can be proposed that PtdIns3K which is induced by PtdIns signaling, and Ca^2+^ release which is induced by both ER stress and PtdIns signaling, are the main factors to induce autophagy and necroptosis in the mutant. As we failed to identify the key regulator of necroptosis in *A. fumigatus*, it is difficult to be absolutely certain whether or not necroptosis was induced simultaneously with autophagy in the mutant. However, it can be concluded that increased necroptosis was associated with autophagy in response to suppression of GPI synthesis in *A. fumigatus*.

## Supporting Information

Figure S1 **Induced expression of genes involved in ribosome assembly in the mutant.** Pathways were analyzed using the SAS pathway enrichment suite (Shanghai biotechnology corporation) using the genes with a fold change of 1.5 or higher. This picture was drawn based on a KEGG pathway. The induced genes are marked with red frames. Blue numbers below the proteins are the locus tag numbers from the *Aspergillus fumigatus* genome.
(TIF)

Figure S2 **Enhanced glycolysis in the mutant.** Pathways were analyzed using the SAS pathway enrichment suite (Shanghai biotechnology corporation) using the genes with a fold change of 1.5 or higher. This picture was drawn based on a KEGG pathway. The enhanced glycolysis pathway is located inside the yellow frame. The differentially regulated genes were labeled with the locus tag descriptions from the annotated *Aspergillus fumigatus* genome. The genes with red background were induced, while the genes with blue background were suppressed in the mutant.
(TIF)

Figure S3 **Enhanced citrate cycle in the mutant.** Pathways were analyzed using the SAS pathway enrichment suite (Shanghai biotechnology corporation) using the genes with a fold change of 1.5 or higher. This picture was drawn based on a KEGG pathway. The enhanced citrate cycle (TCA) is located inside the yellow frame. The differentially regulated genes were labeled with the locus tag descriptions from the annotated *Aspergillus fumigatus* genome. The genes with red background were induced, while the genes with blue background were suppressed in the mutant.
(TIF)

Figure S4 **Enhanced oxidative phosphorylation in the mutant.** Pathways were analyzed using the SAS pathway enrichment suite (Shanghai biotechnology corporation) using the genes with a fold change of 1.5 or higher. This picture was drawn based on a KEGG pathway. The differentially regulated genes were labeled with the locus tag descriptions from the annotated *Aspergillus fumigatus* genome. The genes with red background were induced, while the genes with blue background were suppressed in the mutant.
(TIF)

Figure S5 **Enhanced peroxidation in the mutant.** Pathways were analyzed using the SAS pathway enrichment suite (Shanghai biotechnology corporation) using the genes with a fold change of 1.5 or higher. This picture was drawn based on a KEGG pathway. The differentially regulated genes were labeled with the locus tag descriptions from the annotated *Aspergillus fumigatus* genome. The genes with red background were induced, while the genes with blue background were suppressed in the mutant.
(TIF)

Figure S6 **Biochemical evidence for necroptosis in the mutant.** In A, a Western blotting was carried out using rabbit anti-RIP3 antibody. 50 µg of total protein from the wild-type (WT) or mutant were separated by SDS-PAGE, transferred to a PVDF membrane and detected with anti-RIP3 antibody. As a control, an anti-RAS antibody was used to detect an unrelated protein; In B, 10^3^–10^5^ spores were dotted onto solid CM with or without necrostatin-1 and incubated at 37°C for 36 h.
(TIF)

Table S1 **Primers used for confirmation of the differentially expressed genes.**Primers were designed as described in Materials and Methods. Each primer is 20bp. Their name and sequence were listed in the table.
(DOCX)

Table S2 **Differentially expressed genes (P<0.05, FC>1.5).**The RNA extracted from the mutant grown in CM at 37°C for 36 h was assayed with a genome-wide microarray chip. 3274 genes were differentially expressed (P<0.05), either induced or repressed at least 1.5-fold (Table S2) (NCBI Accession Number: GSE42499).
(XLSX)

Table S3 **Pathway analysis of the differentially expressed genes in the mutant.** Microarray experiments were carried out as described under Materials and Methods. The signal intensities were normalized using Feature Extraction Software (Agilent). Data was analyzed using Genespring Software 5.0. Genes with all signals present (flag = P) were selected for analysis. 1978 genes were selected with P≤0.05, FC≥2.0 by T-test methods. Pathways were analyzed using the SAS pathway enrichment suite (Shanghai biotechnology corporation) using the genes with a fold change of 2 or higher.
(DOCX)

Table S4 **Induced protein translation and degradation in the mutant.** Microarray experiments were carried out as described under Materials and Methods. The signal intensities were normalized using Feature Extraction Software (Agilent). Data was analyzed using Genespring Software 5.0. Genes with all signals present (flag = P) were selected for analysis. Pathways were analyzed using the SAS pathway enrichment suite (Shanghai biotechnology corporation) using the genes with a fold change of 1.5 or higher.
(DOCX)
