# Supplementary figures and images for: Transcriptome and Biochemical Analysis Reveals That Suppression of GPI-Anchor Synthesis Leads to Autophagy and Possible Necroptosis in Aspergillus fumigatus
Source: PLoS One. 2013 Mar 18;8(3):e59013. doi: 10.1371/journal.pone.0059013 (PMC3601126; doi:10.1371/journal.pone.0059013)

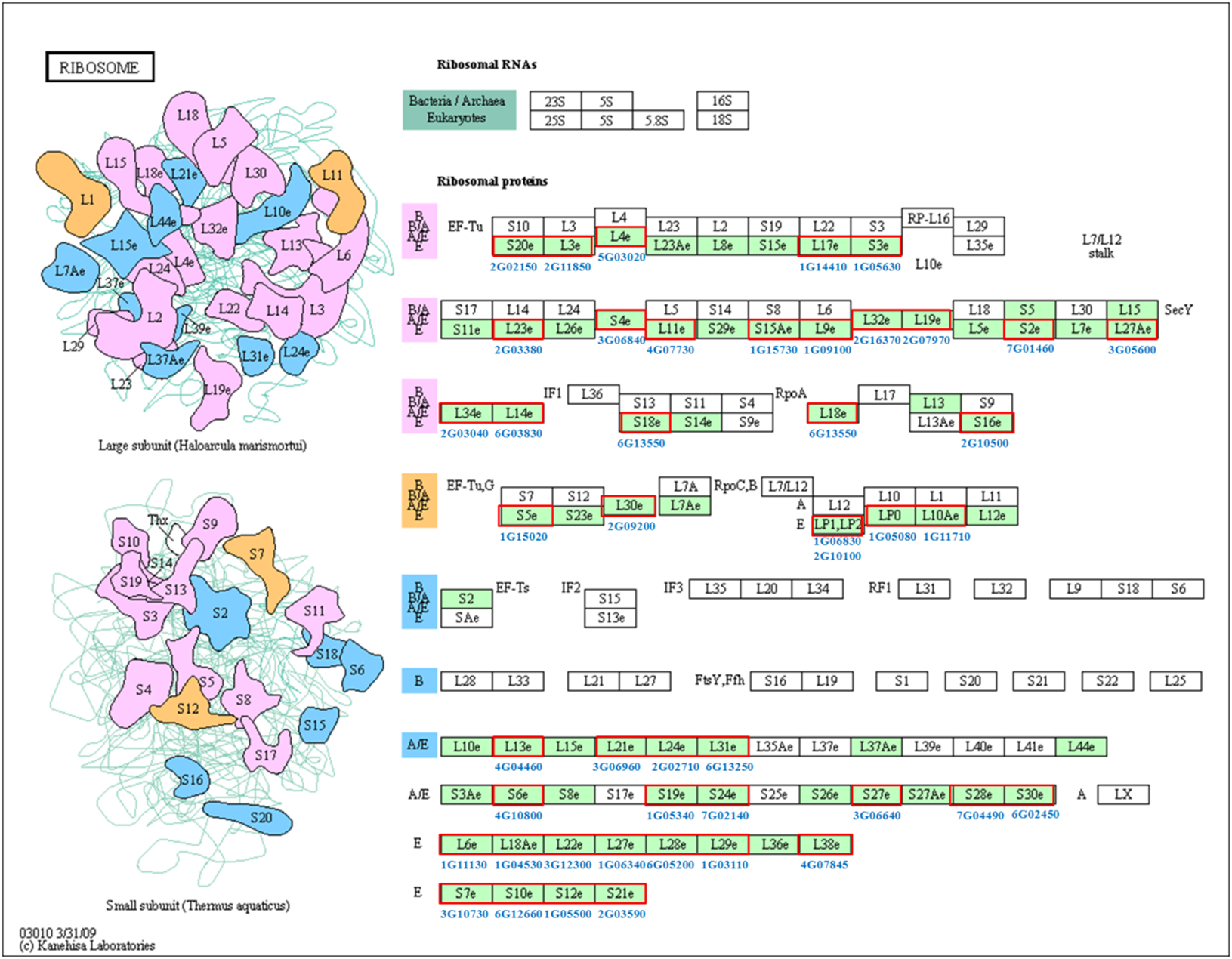

Supplement: Figure S1 — Induced expression of genes involved in ribosome assembly in the mutant. Pathways were analyzed using the SAS pathway enrichment suite (Shanghai biotechnology corporation) using the genes with a fold change of 1.5 or higher. This picture was drawn based on a KEGG pathway. The induced genes are marked with red frames. Blue numbers below the proteins are the locus tag numbers from the Aspergillus fumigatus genome. (TIF) [file pone.0059013.s001.tif]

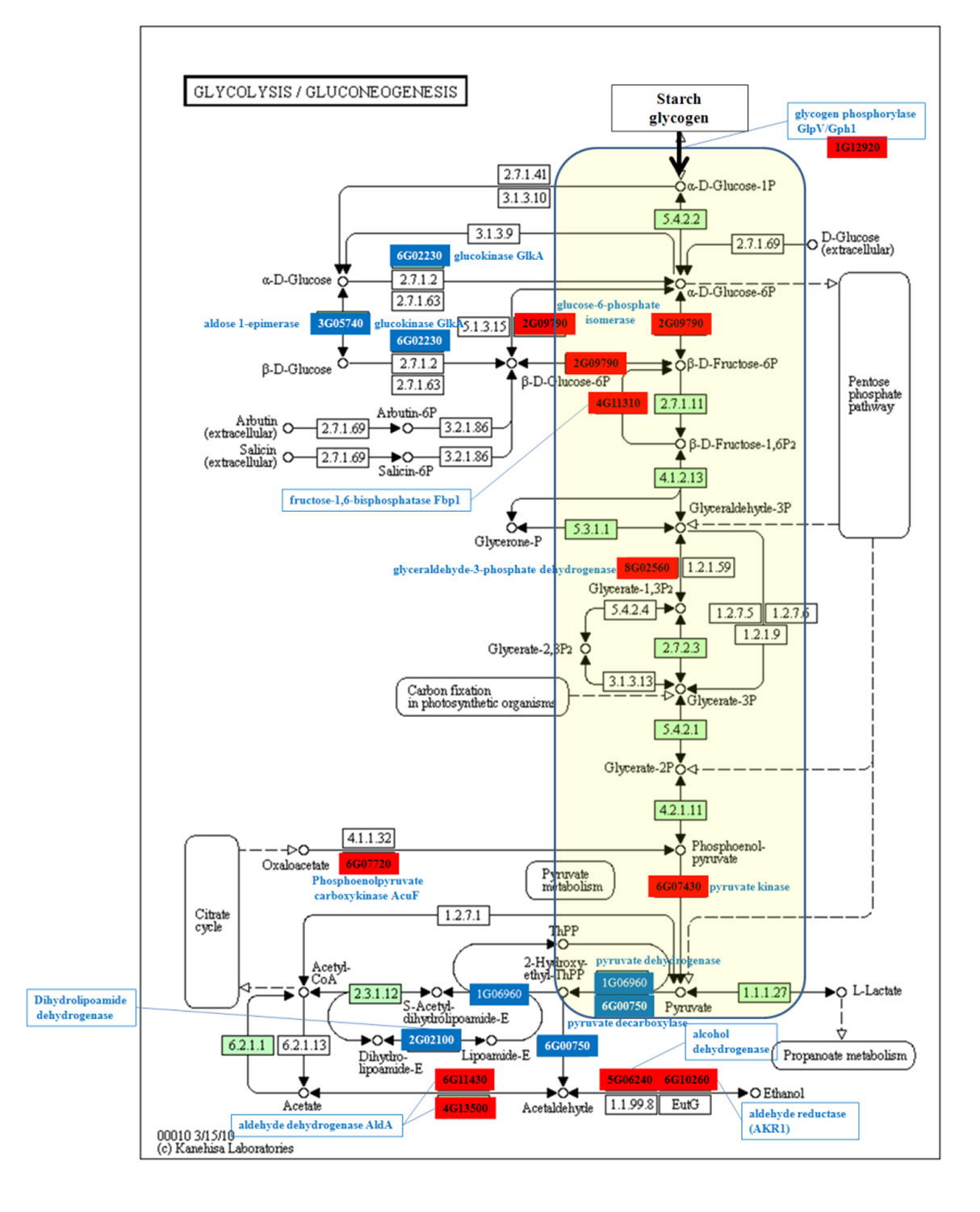

Supplement: Figure S2 — Enhanced glycolysis in the mutant. Pathways were analyzed using the SAS pathway enrichment suite (Shanghai biotechnology corporation) using the genes with a fold change of 1.5 or higher. This picture was drawn based on a KEGG pathway. The enhanced glycolysis pathway is located inside the yellow frame. The differentially regulated genes were labeled with the locus tag descriptions from the annotated Aspergillus fumigatus genome. The genes with red background were induced, while the genes with blue background were suppressed in the mutant. (TIF) [file pone.0059013.s002.tif]

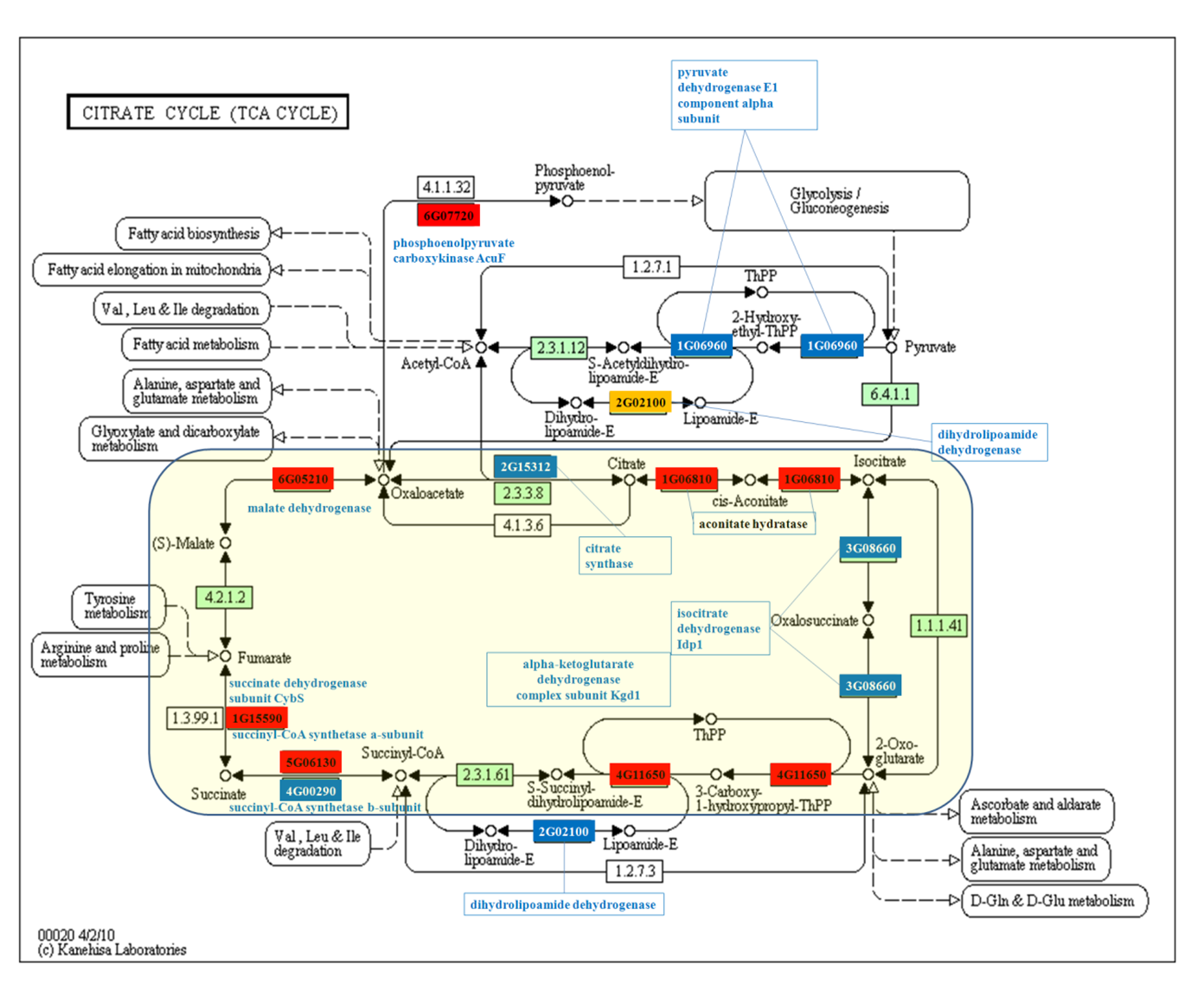

Supplement: Figure S3 — Enhanced citrate cycle in the mutant. Pathways were analyzed using the SAS pathway enrichment suite (Shanghai biotechnology corporation) using the genes with a fold change of 1.5 or higher. This picture was drawn based on a KEGG pathway. The enhanced citrate cycle (TCA) is located inside the yellow frame. The differentially regulated genes were labeled with the locus tag descriptions from the annotated Aspergillus fumigatus genome. The genes with red background were induced, while the genes with blue background were suppressed in the mutant. (TIF) [file pone.0059013.s003.tif]

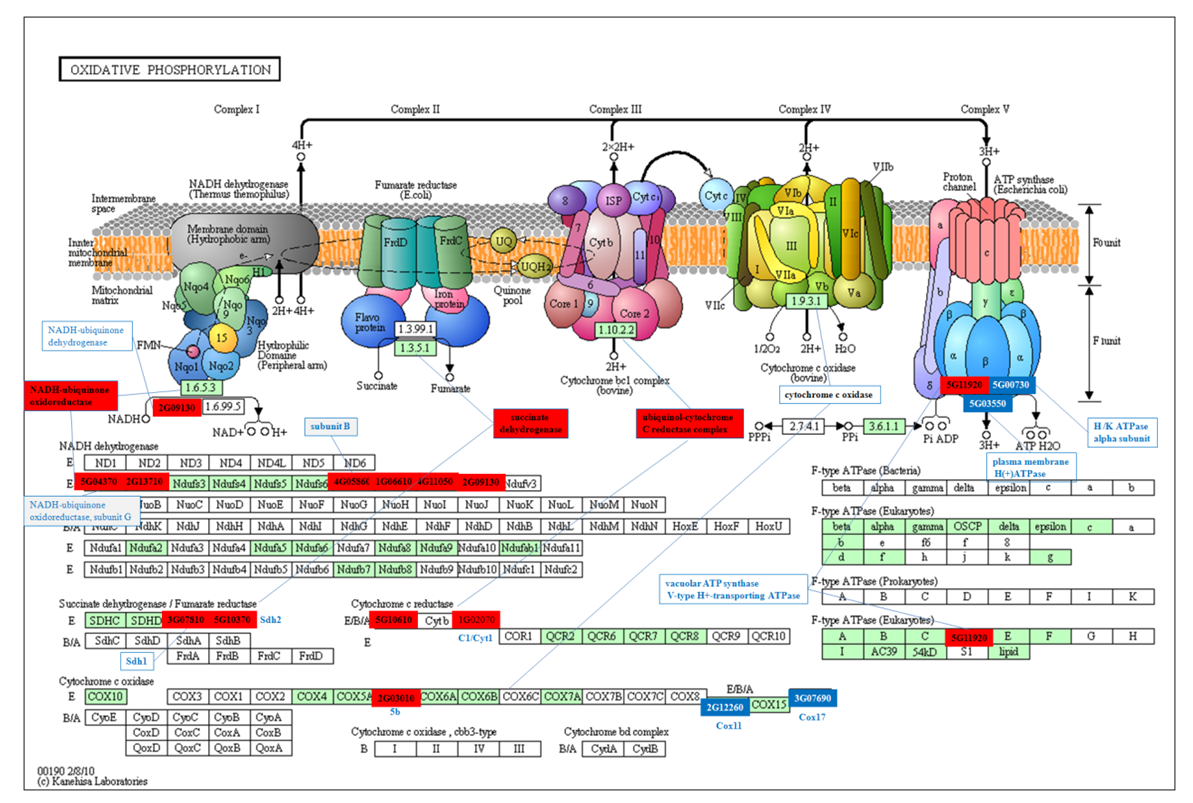

Supplement: Figure S4 — Enhanced oxidative phosphorylation in the mutant. Pathways were analyzed using the SAS pathway enrichment suite (Shanghai biotechnology corporation) using the genes with a fold change of 1.5 or higher. This picture was drawn based on a KEGG pathway. The differentially regulated genes were labeled with the locus tag descriptions from the annotated Aspergillus fumigatus genome. The genes with red background were induced, while the genes with blue background were suppressed in the mutant. (TIF) [file pone.0059013.s004.tif]

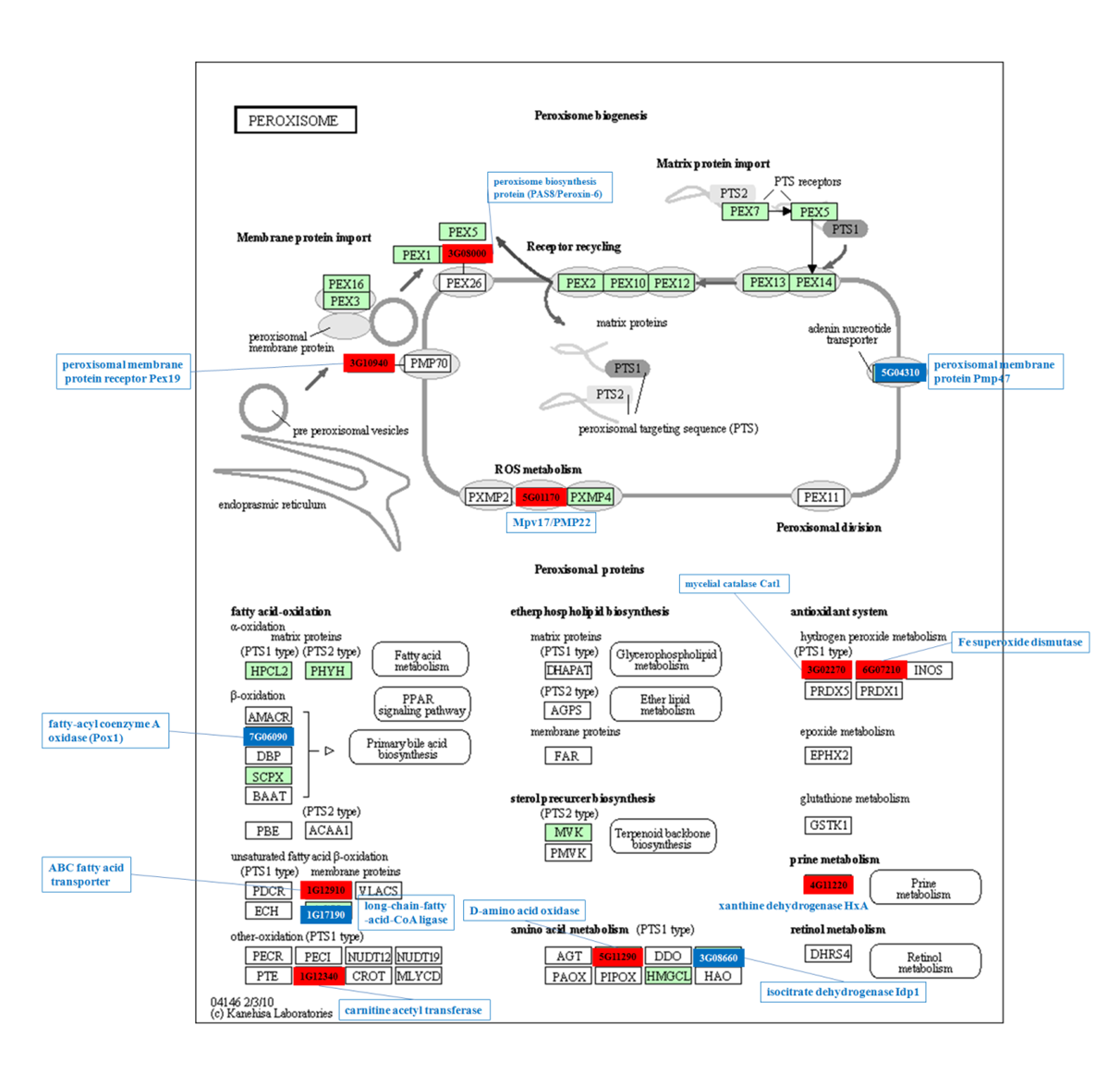

Supplement: Figure S5 — Enhanced peroxidation in the mutant. Pathways were analyzed using the SAS pathway enrichment suite (Shanghai biotechnology corporation) using the genes with a fold change of 1.5 or higher. This picture was drawn based on a KEGG pathway. The differentially regulated genes were labeled with the locus tag descriptions from the annotated Aspergillus fumigatus genome. The genes with red background were induced, while the genes with blue background were suppressed in the mutant. (TIF) [file pone.0059013.s005.tif]

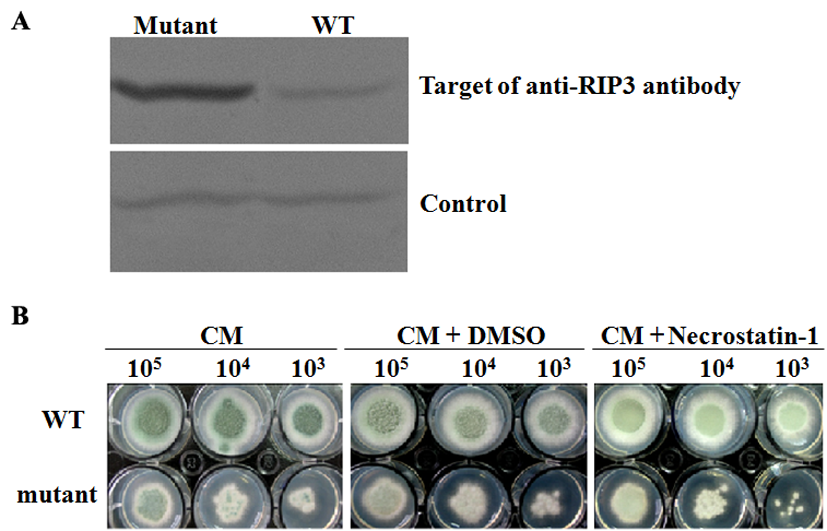

Supplement: Figure S6 — Biochemical evidence for necroptosis in the mutant. In A, a Western blotting was carried out using rabbit anti-RIP3 antibody. 50 µg of total protein from the wild-type (WT) or mutant were separated by SDS-PAGE, transferred to a PVDF membrane and detected with anti-RIP3 antibody. As a control, an anti-RAS antibody was used to detect an unrelated protein; In B, 103–105 spores were dotted onto solid CM with or without necrostatin-1 and incubated at 37°C for 36 h. (TIF) [file pone.0059013.s006.tif]
