# Supplementary material for: Transcriptome and Biochemical Analysis Reveals That Suppression of GPI-Anchor Synthesis Leads to Autophagy and Possible Necroptosis in Aspergillus fumigatus
Source: PLoS One. 2013 Mar 18;8(3):e59013. doi: 10.1371/journal.pone.0059013 (PMC3601126; doi:10.1371/journal.pone.0059013)
Supplement: Table S1 — Primers used for confirmation of the differentially expressed genes.Primers were designed as described in Materials and Methods. Each primer is 20bp. Their name and sequence were listed in the table. (DOCX) [file pone.0059013.s007.docx]

**Table S1.Primers used for confirmation of the differentially expressed genes.**

| Protein | Locus_tag | Primer name | Primer sequence (5´to 3´) |
| --- | --- | --- | --- |
| Hsp70 family HscA | AFUA_8G03930 | 8g039305 | atgattaatgacgccgccaa |
|  |  | 8g039303 | gattcgagctgctgacgaga |
| UBE2G1 | AFUA_6G14130 | 6g141305 | tcccgtttttcacccaaaca |
|  |  | 6g141303 | gactcgtagccgtacttgtc |
| Gel2 | AFUA_6G11390 | 6g113905 | ttacgtccgggcgattcagc |
|  |  | 6g113903 | gacgaatatcggcagcagaa |
| Apg12/Atg12 | AFUA_6G09165 | 6g091655 | gacacagattctgtcttttg |
|  |  | 6g091653 | ccgtcttgaaacatctccat |
| PKC PkcA/Pkc1 | AFUA_5G11970 | 5g119705 | agcagccccactgatacaag |
|  |  | 5g119703 | tgggagagaactgactgcac |
| MAPK Mpkc | AFUA_5G09100 | 5g091005 | gatttcgatgccattgacct |
|  |  | 5g091003 | tagggatatctcagagcgtc |
| PdtIns3K/Vps34 | AFUA_5G08670 | 5g086705 | gacattcgtgtcgaaccgga |
|  |  | 5g086703 | gaaatgccggattgcctcct |
| Aut1/Atg3 | AFUA_5G08170 | 5g081705 | acgcagccaacacgcactta |
|  |  | 5g081703 | agggagaaaggtagccggac |
| MAPK MpkA/Slt2 | AFUA_4G13720 | 4g137205 | accaaagctatcgacgtgtg |
|  |  | 4g137203 | agtcccggcctttgaagaaa |
| S6e | AFUA_4G10800 | 4g108005 | ggaggctgctaacgactacg |
|  |  | 4g108003 | agaagctctccgcttgcgga |
| Calmodulin | AFUA_4G10050 | 4g100505 | tatcggggagaagctcactg |
|  |  | 4g100503 | actcgttgtaatcaatccgg |
| Imp2 | AFUA_3G13840 | 3g138405 | ttacctttcggtcgcctgct |
|  |  | 3g138403 | cggttcacgagtcgtaatcc |
| L22e | AFUA_3G12300 | 3g123005 | caagtacctgaccaagaagt |
|  |  | 3g123003 | cggagctcgtagacaccctt |
| Arp2 | AFUA_2G17560 | 2g175605 | aagagctcgtcgctggagga |
|  |  | 2g175603 | caccttgccaatgtcctcgg |
| PLD | AFUA_2G16520 | 2g165205 | ctaaggtcatgattgccgat |
|  |  | 2g165203 | tgaatcgtgatccccgagct |
| PKA PkaC1 | AFUA_2G12200 | 2g122005 | tatccgtggaggtcagggtg |
|  |  | 2g122003 | atggccatgcacatcttcgc |
| GTP binding protein Bud4 | AFUA_2G08470 | 2g084705 | tgattgcccgtactggcgtc |
|  |  | 2g084703 | ttggcgggtggtttcatggt |
| Rho GTPase ModA/Cdc42 | AFUA_2G05740 | 2g057405 | cgaaggagctgggtgctgta |
|  |  | 2g057403 | cgcaacaatcgcctcatcaa |
| Hsp70 BiP/Kar2 | AFUA_2G04620 | 2g046205 | gatccaagtttatgagggag |
|  |  | 2g046203 | gggagggatacccgtcaact |
| Msg5 | AFUA_2G02760 | 2g027605 | cactgtcgaggatccgctaa |
|  |  | 2g027603 | cttccagcccatccgggaat |
| Gel1 | AFUA_2G01170 | 2g011705 | ggctactcggctgccgatat |
|  |  | 2g011703 | gaagtcactgcgctcgtcgt |
| TilA | AFUA_1G15670 | 1g156705 | actgatattggctgcgcgcc |
|  |  | 1g156703 | gaaaacagtgtcggtggcgt |
| Lsm7 | AFUA_1G14290 | 1g142905 | tggaggacgagaggttagtg |
|  |  | 1g142903 | accacgcatgctttccttga |
| Plc1 | AFUA_1G13250 | 1g132505 | aactgagcagtctgtctcag |
|  |  | 1g132503 | tctgcagaatagggtggaga |
| QutG | AFUA_1G11600 | 1g116005 | taaggagtctggggagtgcg |
|  |  | 1g116003 | ccacatcccactcccaacaa |
| GRR1 | AFUA_1G05970 | 1g059705 | gacagtgccaggaatacatg |
|  |  | 1g059703 | cggatccaagaaagcctgca |
| Apg6/Atg6 | AFUA_8G05170 | 8g051705 | ggagaattcgtggagaagac |
|  |  | 8g051703 | gtacgggagttttaaccctg |
| Vps15 | AFUA_6G11190 | 6g111905 | agacattgtttcggaaggag |
|  |  | 6g111903 | atgtgttctgtagtcacgag |
| Apg5/Atg5 | AFUA_6G07040 | 6g070405 | gtgcaagacgtggatctcct |
|  |  | 6g070403 | aaagcggattgggacgttgc |
| Vac8 | AFUA_5G13540 | 5g135405 | acctctcctcaaaagttggc |
|  |  | 5g135403 | ggaaccttttgagataaccg |
| Pdd7/Atg1 | AFUA_4G09050 | 4g090505 | gataaagctgctgacgatcc |
|  |  | 4g090503 | cgtttcgcaagaatcgccag |
| Atg4 | AFUA_3G05340 | 3g053405 | attccgatttctggtagggg |
|  |  | 3g053403 | ggttcattcgcgacgagaat |
| Atg17 | AFUA_2G14100 | 2g141005 | aagacagcaaatcgccttgc |
|  |  | 2g141003 | gaaagcctgctccacgagtt |
| ApgG/Atg11 | AFUA_2G06250 | 2g062505 | gccgatgaactctgtaagag |
|  |  | 2g062503 | accgccaaggctgacgctat |
| Aut7/Atg8 | AFUA_1G07470 | 1g074705 | atgaggtcctgccaccaacc |
|  |  | 1g074703 | gccagagtatgtgatgtaca |
| calcineurin catalytic subunit CnaA | AFUA_5G09360 | 5g093605 | gaaagacgggctgccctcga |
|  |  | 5g093603 | gagccggcggacatgctaat |
| AP-2 adaptor complex subunit | AFUA_5G07930 | 5g079305 | gtcgccatcaaggcgaacta |
|  |  | 5g079303 | ggtcgtcttggccgtgttca |
| phosphatase family protein | AFUA_5G07860 | 5g078605 | tcctaccgcagctgaaacag |
|  |  | 5g078603 | gcttgaaggctcgataagct |
| TOR pathway TorA | AFUA_2G10270 | 2g1027052 | tctggaagcacattcacgat |
|  |  | 2g1027032 | taacggacgtaatggactgt |
| bZIP transcription factor HacA | AFUA_3G04070 | haca5 | cttcgcttcagttgtagagtc |
|  |  | haca3 | gtctttgtctcctgcactttg |
| protein disulfide isomerase Pdi1, putative | AFUA_2G06150 | pdia5 | ttccgatgtcgtctctctaac |
|  |  | pdia3 | tatttaggagctagagccttg |
| disulfide isomerase (TigA), putative | AFUA_5G12260 | tiga5 | tcacactgctcgtcggtattg |
|  |  | tiga3 | aggggcgaagaattcaacaag |
