# Supplementary material for: Transcriptome and Biochemical Analysis Reveals That Suppression of GPI-Anchor Synthesis Leads to Autophagy and Possible Necroptosis in Aspergillus fumigatus
Source: PLoS One. 2013 Mar 18;8(3):e59013. doi: 10.1371/journal.pone.0059013 (PMC3601126; doi:10.1371/journal.pone.0059013)
Supplement: Table S3 — Pathway analysis of the differentially expressed genes in the mutant. Microarray experiments were carried out as described under Materials and Methods. The signal intensities were normalized using Feature Extraction Software (Agilent). Data was analyzed using Genespring Software 5.0. Genes with all signals present (flag = P) were selected for analysis. 1978 genes were selected with P≤0.05, FC≥2.0 by T-test methods. Pathways were analyzed using the SAS pathway enrichment suite (Shanghai biotechnology corporation) using the genes with a fold change of 2 or higher. (DOCX) [file pone.0059013.s009.docx]

**Table S3. Pathway analysis of the differentially expressed genes in the mutant.**

| **PathwayDB** | **Pathway** | **Hits** | **Total** | **Percent** | **Enrichment test pvalue** |
| --- | --- | --- | --- | --- | --- |
| Kegg | Biosynthesis of unsaturated fatty acids | 10 | 16 | 62.50% | 0.0064 |
| Kegg | Starch and sucrose metabolism | 28 | 78 | 35.90% | 0.007 |
| Kegg | Glyoxylate and dicarboxylate metabolism | 11 | 20 | 55.00% | 0.0087 |
| Kegg | Pyruvate metabolism | 17 | 42 | 40.48% | 0.0134 |
| Kegg | Propanoate metabolism | 17 | 44 | 38.64% | 0.0187 |
| Kegg | Citrate cycle (TCA cycle) | 13 | 30 | 43.33% | 0.0193 |
| Kegg | Tyrosine metabolism | 24 | 74 | 32.43% | 0.0291 |
| Kegg | Penicillin and cephalosporin biosynthesis | 4 | 4 | 100.00% | 0.0304 |
| Kegg | Butanoate metabolism | 28 | 92 | 30.43% | 0.0356 |
| Kegg | Pentose and glucuronate interconversions | 8 | 17 | 47.06% | 0.044 |
| Kegg | Linoleic acid metabolism | 17 | 50 | 34.00% | 0.0443 |
| Kegg | Galactose metabolism | 13 | 35 | 37.14% | 0.0458 |
| Kegg | Phenylalanine metabolism | 15 | 43 | 34.88% | 0.0487 |
| Kegg | Phosphatidylinositol signaling system | 6 | 11 | 54.55% | 0.0498 |
| Kegg | Sphingolipid metabolism | 10 | 25 | 40.00% | 0.054 |
| Kegg | Metabolic pathways | 199 | 884 | 22.51% | 0.0588 |
| Kegg | D-Arginine and D-ornithine metabolism | 3 | 3 | 100.00% | 0.0619 |
| Kegg | Cyanoamino acid metabolism | 9 | 26 | 34.62% | 0.1146 |
| Kegg | Limonene and pinene degradation | 29 | 112 | 25.89% | 0.1264 |
| Kegg | Glycerolipid metabolism | 10 | 31 | 32.26% | 0.1314 |
| Kegg | Fatty acid biosynthesis | 4 | 9 | 44.44% | 0.157 |
| Kegg | Glycolysis / Gluconeogenesis | 15 | 54 | 27.78% | 0.1625 |
| Kegg | Folate biosynthesis | 5 | 13 | 38.46% | 0.1669 |
| Kegg | Other glycan degradation | 5 | 13 | 38.46% | 0.1669 |
| Kegg | Arachidonic acid metabolism | 3 | 6 | 50.00% | 0.1773 |
| Kegg | Glycosphingolipid biosynthesis - globo series | 3 | 7 | 42.86% | 0.2237 |
| Kegg | Sulfur metabolism | 5 | 15 | 33.33% | 0.2296 |
| Kegg | Arginine and proline metabolism | 13 | 50 | 26.00% | 0.24 |
| Kegg | Glutathione metabolism | 8 | 28 | 28.57% | 0.2403 |
| Kegg | Fructose and mannose metabolism | 21 | 88 | 23.86% | 0.264 |
| Kegg | Metabolism of xenobiotics by cytochrome P450 | 7 | 26 | 26.92% | 0.3037 |
| Kegg | Nicotinate and nicotinamide metabolism | 3 | 9 | 33.33% | 0.3212 |
| Kegg | Thiamine metabolism | 3 | 9 | 33.33% | 0.3212 |
| Kegg | Histidine metabolism | 10 | 41 | 24.39% | 0.3375 |
| Kegg | Amino sugar and nucleotide sugar metabolism | 18 | 79 | 22.78% | 0.3444 |
| Kegg | beta-Alanine metabolism | 6 | 23 | 26.09% | 0.3494 |
| Kegg | Tryptophan metabolism | 9 | 37 | 24.32% | 0.3534 |
| Kegg | Inositol phosphate metabolism | 5 | 19 | 26.32% | 0.3686 |
| Kegg | Cysteine and methionine metabolism | 8 | 33 | 24.24% | 0.3711 |
| Kegg | Fatty acid metabolism | 8 | 33 | 24.24% | 0.3711 |
| Kegg | Methane metabolism | 6 | 24 | 25.00% | 0.3813 |
| Kegg | alpha-Linolenic acid metabolism | 2 | 6 | 33.33% | 0.3942 |
| Kegg | Lysine biosynthesis | 3 | 11 | 27.27% | 0.419 |
| Kegg | Ascorbate and aldarate metabolism | 2 | 7 | 28.57% | 0.4561 |
| Kegg | Glycine, serine and threonine metabolism | 11 | 51 | 21.57% | 0.4591 |
| Kegg | Peroxisome | 9 | 43 | 20.93% | 0.5052 |
| Kegg | Taurine and hypotaurine metabolism | 3 | 13 | 23.08% | 0.5116 |
| Kegg | Ether lipid metabolism | 2 | 8 | 25.00% | 0.5142 |
| Kegg | Lipoic acid metabolism | 1 | 3 | 33.33% | 0.5169 |
| Kegg | Riboflavin metabolism | 6 | 29 | 20.69% | 0.5376 |
| Kegg | Valine, leucine and isoleucine degradation | 8 | 40 | 20.00% | 0.5574 |
| Kegg | Synthesis and degradation of ketone bodies | 2 | 9 | 22.22% | 0.5681 |
| Kegg | RNA polymerase | 5 | 25 | 20.00% | 0.5737 |
| Kegg | Steroid biosynthesis | 4 | 21 | 19.05% | 0.6167 |
| Kegg | Ubiquinone and other terpenoid-quinone biosynthesis | 2 | 10 | 20.00% | 0.6174 |
| Kegg | Alanine, aspartate and glutamate metabolism | 6 | 32 | 18.75% | 0.6228 |
| Kegg | Glycerophospholipid metabolism | 7 | 38 | 18.42% | 0.6386 |
| Kegg | Pantothenate and CoA biosynthesis | 3 | 18 | 16.67% | 0.703 |
| Kegg | Terpenoid backbone biosynthesis | 2 | 13 | 15.38% | 0.7393 |
| Kegg | Lysine degradation | 4 | 26 | 15.38% | 0.759 |
| Kegg | Pentose phosphate pathway | 4 | 26 | 15.38% | 0.759 |
| Kegg | Base excision repair | 3 | 20 | 15.00% | 0.7615 |
| Kegg | Vitamin B6 metabolism | 1 | 7 | 14.29% | 0.7667 |
| Kegg | One carbon pool by folate | 2 | 14 | 14.29% | 0.7718 |
| Kegg | Purine metabolism | 14 | 84 | 16.67% | 0.7731 |
| Kegg | Aminoacyl-tRNA biosynthesis | 6 | 39 | 15.38% | 0.7819 |
| Kegg | Biotin metabolism | 1 | 8 | 12.50% | 0.8055 |
| Kegg | Porphyrin and chlorophyll metabolism | 2 | 16 | 12.50% | 0.8264 |
| Kegg | Ribosome | 11 | 72 | 15.28% | 0.8356 |
| Kegg | Protein export | 2 | 17 | 11.76% | 0.849 |
| Kegg | Selenoamino acid metabolism | 4 | 31 | 12.90% | 0.8566 |
| Kegg | Valine, leucine and isoleucine biosynthesis | 3 | 25 | 12.00% | 0.8675 |
| Kegg | Phenylalanine, tyrosine and tryptophan biosynthesis | 3 | 26 | 11.54% | 0.8828 |
| Kegg | Nitrogen metabolism | 3 | 27 | 11.11% | 0.8966 |
| Kegg | Regulation of autophagy | 1 | 12 | 8.33% | 0.9061 |
| Kegg | Pyrimidine metabolism | 8 | 62 | 12.90% | 0.9145 |
| Kegg | Basal transcription factors | 2 | 22 | 9.09% | 0.9266 |
| Kegg | Oxidative phosphorylation | 9 | 75 | 12.00% | 0.9529 |
| Kegg | MAPK signaling pathway | 2 | 26 | 7.69% | 0.9597 |
| Kegg | Glycosylphosphatidylinositol(GPI) anchor biosynthesis | 1 | 18 | 5.56% | 0.9685 |
| Kegg | Nucleotide excision repair | 3 | 39 | 7.69% | 0.9791 |
| Kegg | RNA degradation | 3 | 43 | 6.98% | 0.9882 |
| Kegg | Endocytosis | 2 | 35 | 5.71% | 0.9901 |
| Kegg | Ubiquitin mediated proteolysis | 3 | 52 | 5.77% | 0.9968 |
| Kegg | DNA replication | 1 | 33 | 3.03% | 0.998 |
| Kegg | Cell cycle | 3 | 70 | 4.29% | 0.9998 |
| Kegg | Spliceosome | 4 | 83 | 4.82% | 0.9999 |
| Kegg | Meiosis | 1 | 54 | 1.85% | 1 |

Microarray experiments were carried out as described under Materials and Methods. The signal intensities were normalized using Feature Extraction Software (Agilent). Data was analyzed using Genespring Software 5.0. Genes with all signals present (flag=P) were selected for analysis. 1978 genes were selected with P≤0.05, FC≥2.0 by T-test methods. Pathways were analyzed using the SAS pathway enrichment suite (Shanghai biotechnology corporation) using the genes with a fold change of 2 or higher.
