# Supplementary material for: Transcriptome and Biochemical Analysis Reveals That Suppression of GPI-Anchor Synthesis Leads to Autophagy and Possible Necroptosis in Aspergillus fumigatus
Source: PLoS One. 2013 Mar 18;8(3):e59013. doi: 10.1371/journal.pone.0059013 (PMC3601126; doi:10.1371/journal.pone.0059013)
Supplement: Table S4 — Induced protein translation and degradation in the mutant. Microarray experiments were carried out as described under Materials and Methods. The signal intensities were normalized using Feature Extraction Software (Agilent). Data was analyzed using Genespring Software 5.0. Genes with all signals present (flag = P) were selected for analysis. Pathways were analyzed using the SAS pathway enrichment suite (Shanghai biotechnology corporation) using the genes with a fold change of 1.5 or higher. (DOCX) [file pone.0059013.s010.docx]

**Table S4. Induced protein translation and degradation in the mutant.**

| Pathway | Locus tag | Protein | Up-regulation (fold) |
| --- | --- | --- | --- |
| Protein translation | AFUA_2G01920 | glutaminyl-tRNA synthetase | 11.5 |
|  | AFUA_5G10640 | tyrosyl-tRNA synthetase | 2.2 |
|  | AFUA_1G09010 | methionyl-tRNA synthetase | 2.5 |
|  | AFUA_8G07110 | alanyl-tRNA synthetase | 1.6 |
|  | AFUA_2G01640 | tryptophanyl-tRNA synthetase | 1.8 |
|  | AFUA_1G13710 | isoleucyl-tRNA synthetase | 1.7 |
|  | AFUA_6G12630 | leucyl-tRNA synthetase | 1.7 |
|  | AFUA_5G09610 | cysteinyl-tRNA synthetase | 2.8 |
|  | AFUA_4G12920 | histidyl-tRNA synthetase | 1.4 |
|  | AFUA_2G02590 | aspartyl-tRNA synthetase Dps1 | 1.9 |
|  | AFUA_5G05490 | seryl-tRNA synthetase | 2.1 |
|  | AFUA_4G13700 | threonyl-tRNA synthetase | 1.7 |
|  | AFUA_2G03580 | phenylalanyl-tRNA synthetase | 1.6 |
|  | AFUA_5G05920 | glycyl-tRNA synthetase | 1.6 |
| Proteasome | AFUA_3G08940 | proteasome regulatory particle subunit RpnL | 1.6 |
|  | AFUA_2G15070 | 26S proteasome regulatory subunit S5A | 1.5 |
|  | AFUA_3G11300 | proteasome component Prs2 | 1.7 |
|  | AFUA_7G05870 | proteasome component Pre8 | 1.6 |
|  | AFUA_6G08960 | proteasome component Pre9 | 1.6 |
|  | AFUA_5G02150 | proteasome component Pre6 | 1.8 |
|  | AFUA_4G07510 | proteasome component Pre1 | 2.0 |
|  | AFUA_5G10740 | 20S proteasome maturation protein Ump1 | 1.6 |
| Ubiquitin-mediated proteolysis | AFUA_6G13170 | ubiquitin conjugating enzyme (UbcK) | 1.9 |
|  | AFUA_6G14130 | ubiquitin conjugating enzyme | 3.0 |
|  | AFUA_5G04060 | ubiquitin conjugating enzyme (UbcH) | 1.7 |
|  | AFUA_6G09160 | ubiquitin conjugating enzyme Ubc8 | 1.9 |
|  | AFUA_5G13510 | cell cycle control protein (Cwf8) | 1.7 |
|  | AFUA_1G05970 | ubiquitin ligase complex F-box protein GRR1 | 3.2 |
|  | AFUA_1G14730 | cell division cycle protein Cdc20 | 1.6 |
|  | AFUA_1G15710 | anaphase-promoting complex component Cut20/Apc4 | 1.7 |
|  | AFUA_5G02440 | 20S cyclosome subunit (APC8) | 1.5 |
|  | AFUA_6G08220 | SCF ubiquitin ligase subunit CulC | 1.7 |

Microarray experiments were carried out as described under Materials and Methods. The signal intensities were normalized using Feature Extraction Software (Agilent). Data was analyzed using Genespring Software 5.0. Genes with all signals present (flag=P) were selected for analysis. Pathways were analyzed using the SAS pathway enrichment suite (Shanghai biotechnology corporation) using the genes with a fold change of 1.5 or higher.
